# Multiscale Design of Dental Restorative Materials: Mechanistic Foundations, Technological Innovations, and Clinical Translation

**DOI:** 10.1002/advs.202511258

**Published:** 2025-11-11

**Authors:** Bailei Li, Ruixue Ai, Xinjiani Chen, Yaning Wang, Chenlei Pan, Suxian Song, Chenjie Yang, Yu Zhou, Zhen Zhang, Xiaojun Liu, Xiaodi Liu, Liping Yao, Quanbao Zhang, Wenxue Gu, Rongqing Zhang

**Affiliations:** ^1^ Department of Biotechnology and Biomedicine Yangtze Delta Region Institute of Tsinghua University Jiaxing Zhejiang 314006 China; ^2^ Zhejiang Key Laboratory of Multiomics and Molecular Enzymology Yangtze Delta Region Institute of Tsinghua University Jiaxing Zhejiang 314006 China; ^3^ Department of Clinical Molecular Biology University of Oslo and Akershus University Hospital Lørenskog 1478 Norway; ^4^ Department of Pharmaceutics Jiaxing Key Laboratory for Photonanomedicine and Experimental Therapeutics College of Medicine Jiaxing University Jiaxing Zhejiang 314001 China; ^5^ Southwest Jiaotong University Chengdu Sichuan 611756 China; ^6^ Ministry of Education Key Laboratory of Protein Sciences School of Life Sciences Tsinghua University Beijing 100084 China

**Keywords:** AI‐driven material discovery, bioinspired dental materials, clinical translation, multiscale biomaterial design, smart responsive interfaces

## Abstract

Dental restorative materials remain constrained by clinical unmet needs in biomechanical durability, bioactivity, and long‐term tissue integration, despite incremental advances in conventional ceramics and polymers. Addressing these gaps requires functional innovation. Multiscale design strategies bridge atomic‐level material engineering with macroscopic clinical performance. This review highlights transformative approaches, including bioinspired architectures (e.g., decellularized extracellular matrix scaffolds) that replicate native oral tissue mechanics while promoting cell‐driven remodeling, and smart interfaces (e.g., pH‐responsive polymers) for dynamic biofilm suppression. Functional enhancements are achieved via 3D printing for anatomically precise restorations, nanotechnology for crack‐resistant ceramics, and surface functionalization (e.g., graphene oxide coatings) to accelerate osseointegration. Emerging paradigms such as 4D‐printed shape‐memory composites and artificial intelligence (AI)‐driven inverse design further exemplify innovation by enabling self‐adapting materials and accelerated discovery of bioactive formulations. Critically, barriers are analyzed to clinical translation, including scalable manufacturing of hierarchical structures, mitigating biodegradation in aggressive oral environments, and balancing cost‐effectiveness with performance. By integrating mechanistic insights with clinical translation, this work provides a blueprint for next‐generation functional dental materials that reconcile laboratory innovation with real‐world clinical demands.

## Graphical abstract

From conventional ceramics to bioinspired smart composites, this review charts the evolution of dental restorative biomaterials. Integrating materials innovation, advanced manufacturing technologies, and bioinspired strategies, it presents a roadmap for developing functional, clinically translatable restorations that combine durability, adaptability, and biological integration. These aim to create next‐generation solutions capable of sustaining long‐term oral health and enhancing patient quality of life.

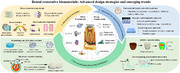

## 1 Introduction

Dental restorative materials are critical for mitigating the functional and aesthetic consequences of traumatic injuries, dental caries, and chronic wear, which collectively affect over 3.5 billion individuals globally and account for 5–10% of healthcare expenditures in developed nations.^[^ ^1^, ^2^ ^]^ However, clinical unmet needs persist: conventional resin‐based composites (RBCs) exhibit a 30–50% failure rate within a decade due to secondary caries, wear‐induced morphology loss (>20 µm/year), and marginal leakage from polymerization shrinkage (>2% volumetric contraction).^[^ ^3^, ^4^, ^5^, ^6^ ^]^ These limitations are exacerbated in patients with systemic conditions (e.g., diabetes) or compromised oral hygiene, where biofilm‐driven degradation accelerates material failure.^[^ ^7^, ^8^, ^9^, ^10^ ^]^ Critically, the fundamental mismatch between synthetic materials and the dentinoenamel complex—a hierarchical biological system optimized over 500 million years of evolution—has hindered progress. Natural teeth achieve exceptional durability through multiscale damage‐redistribution mechanisms, including graded mineralization (20 nm hydroxyapatite crystallites to 100 µm dentinal tubules) and collagen‐mineral interfaces that dissipate crack energy.^[^ ^11^, ^12^ ^]^ Replicating such functionality demands a paradigm shift from empirical material selection to function‐driven multiscale design.

**Figure 1:**
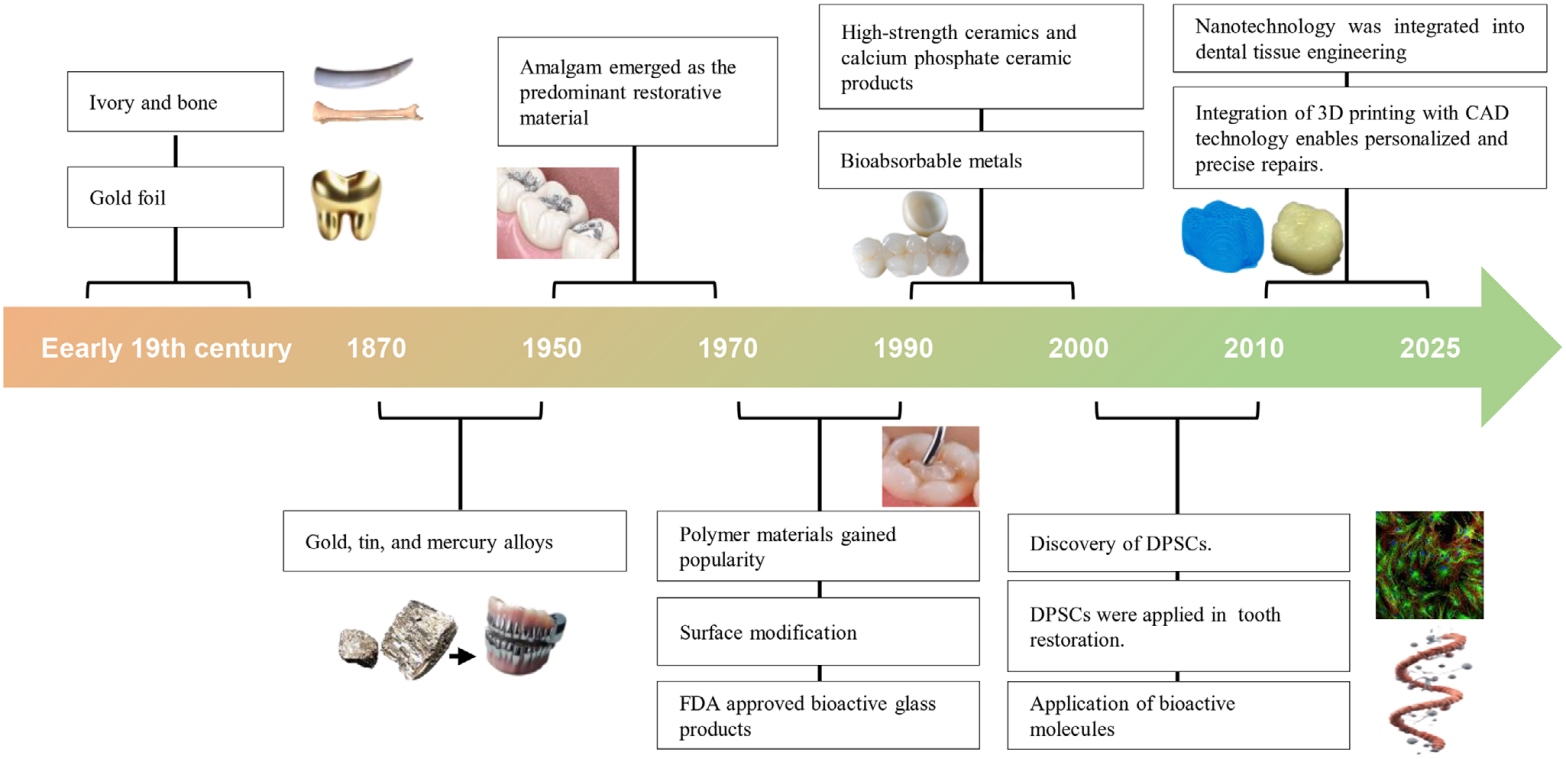
Timeline of main milestones in biomaterials design for dental restoration. Initial dental restorative efforts utilized ivory and gold foil. Subsequently, metal and alloy materials became widely used, followed by the exploration of calcium phosphate ceramics and bioabsorbable metals, drawing insights from bone repair techniques. The advent of polymer materials has led to significant breakthroughs in dental restoration, and the incorporation of bioglass has injected new vitality. In addition to exploring materials science, attention is also paid to the application of biologically active molecules. DPSCs: dental pulp stem cells; FDA: United States Food and Drug Administration.

Recent advances in materials science and additive manufacturing have opened transformative pathways. The evolution of dental restorative materials has been inextricably linked to humanity's pursuit of functional oral rehabilitation. From the metallurgical ingenuity of ancient civilizations to today's nanotechnology‐driven innovations, this field has undergone paradigm shifts driven by interdisciplinary convergence and clinical demands (**Figure** **1**). Bioinspired engineering has produced hydroxyapatite/polymer composites that mimic enamel's staggered platelet architecture. These composites achieve fracture energies (3.5 kJ m^−^^2^), ≈40% higher than conventional RBCs.^[^ ^13^, ^14^, ^15^ ^]^ Similarly, 4D‐printed shape‐memory polymers now enable self‐adapting restorations that compensate for thermal/mechanical stresses in situ, addressing the long‐standing challenge of interfacial debonding.^[^ ^16^, ^17^, ^18^, ^19^, ^20^, ^21^, ^22^ ^]^ On the manufacturing front, laser‐assisted metal deposition integrates 50 µm scale sensors into crowns, enabling real‐time monitoring of occlusal forces and biofilm accumulation, a milestone toward “cyber‐physical” dental prosthetics.^[^ ^23^, ^24^ ^]^ Meanwhile, nanoscale phase‐change fillers, inspired by smart construction materials, have produced RBCs with synchronized thermomechanical/optical properties, resolving the aesthetic‐durability trade‐off.^[^ ^25^, ^26^ ^]^

Despite these breakthroughs, clinical translation remains bottlenecked by fragmented innovation ecosystems. For instance, while multimaterial 3D printing permits anatomical customization, residual stresses from layer‐by‐layer fabrication (up to 120 MPa) compromise dentin adhesion.^[^ ^27^, ^28^, ^29^ ^]^ Likewise, antimicrobial coatings degrade under cyclic masticatory loads (10⁶–10⁷ cycles per year), losing efficacy within 2–3 years.^[^ ^30^, ^31^ ^]^ These gaps underscore the need for systems‐level design that co‐optimizes: 1) biomimetic hierarchical structures (e.g., enamel‐like graded stiffness), 2) scalable manufacturing protocols (e.g., stress‐relieved 3D printing), and 3) dynamic adaptation to the oral environment (e.g., pH‐responsive self‐healing).

This review deconstructs the evolutionary design principles of natural teeth, analyzing how their nanoscale‐to‐macroscale architecture confers resilience. We then evaluate emerging fabrication technologies through a clinical‐translation lens, assessing how parameters like laser sintering density or voxel‐level composition control impact long‐term performance. By integrating mechanistic insights (e.g., collagen‐mineral energy dissipation) with industry‐scalable solutions (e.g., artificial intelligence (AI)‐optimized print paths), we propose a roadmap for next‐generation “bio‐smart” restoratives that transcend passive replacement to actively participate in oral homeostasis. This framework bridges materials innovation with clinical realities, offering a blueprint for functional dental materials that are both biologically inspired and translationally robust.

## 2 Dental Tissue Structure and Implications for Restorative Biomaterials Design

**Figure 2:**
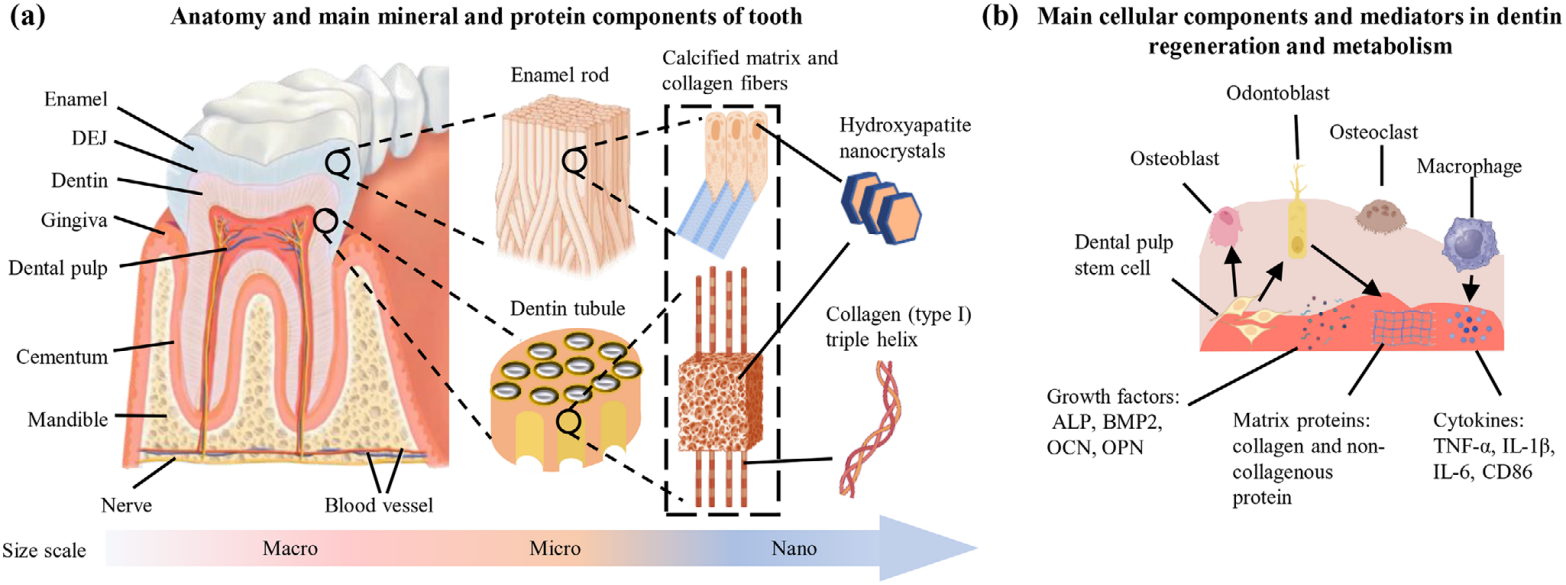
Bionic physiology of candidate biomaterials for dental defect repair. ALP: alkaline phosphatase; BMP2: bone morphogenetic protein type II receptor; OCN: osteocalcin; OPN: osteopontin; TNF‐α: tumor necrosis factor‐alpha; IL‐1β: interleukin‐1β; IL‐6: interleukin‐6; CD86: leukocyte differentiation antigen 86.

The intricate hierarchical architecture of natural dental tissues, namely enamel, dentin, cementum, and their interfaces, is the result of millions of years of evolutionary optimization. This architecture provides invaluable insights for the design of next‐generation restorative biomaterials. From the nanoscale to the macroscale (**Figure** **2**a), these tissues display precisely coordinated structural features that work in synergy to confer mechanical resilience, bioactive functionality, and dynamic adaptability–attributes that current synthetic restoratives find challenging to replicate.

Enamel exhibits exceptional wear resistance due to its highly ordered prismatic architecture. Here, hydroxyapatite (HAP) crystallites (accounting for 96 wt%) are assembled into interlocking bundles with a diameter of 4–8 µm and a controlled crystallographic orientation. This unique microstructure gives rise to anisotropic mechanical properties. The surface hardness, measured by the Vickers method, reaches 3–5 GPa and gradually decreases to ≈1 GPa near the dentinoenamel junction (DEJ).^[^ ^32^, ^33^, ^34^ ^]^ Such hardness gradients facilitate efficient stress redistribution, while crack deflection at prism boundaries enhances fracture toughness.^[^ ^35^, ^36^ ^]^ Modern composites, however, fail to fully emulate this design principle because of difficulties in achieving over 90% HAP alignment while maintaining optical properties (with a refractive index mismatch of less than 0.05).^[^ ^37^, ^38^ ^]^

Beneath the enamel, dentin features an ingenious hybrid structure. It combines type I collagen fibrils with a diameter of 50 nm and intrafibrillar HAP nanoparticles, creating a viscoelastic masterpiece. This structure is capable of dissipating ≈30% of occlusal energy through sophisticated mechanisms, including collagen unfolding and sacrificial bond rupture.^[^ ^39^ ^]^ Its tubular microstructure, with a density of 15 000—45 000 tubules per square meter, enables fluid‐mediated stress redistribution. Importantly, dentin's regenerative capacity relies on odontoblasts lining the pulp chamber, which secrete reactionary dentin in response to injury.^[^ ^40^ ^]^ These cells are regulated by signaling molecules such as transforming growth factor‐β (TGF‐β) and bone morphogenetic proteins (BMPs), which orchestrate stem cell recruitment (e.g., DPSCs) and matrix mineralization (Figure 2b).^[^ ^41^ ^]^ Wnt/β‐catenin pathways further modulate odontoblast differentiation, highlighting the need for restoratives to preserve or enhance these bioactive cues.^[^ ^42^ ^]^ As a result, the elastic modulus varies spatially, ranging from 20 GPa near the DEJ to 10–12 GPa internally.^[^ ^43^, ^44^ ^]^ Current dentin bonding systems have a fundamental misunderstanding of this dynamic system. Hydrophobic adhesives that occlude dentinal tubules disrupt the natural fluid dynamics, leading to interfacial failure under cyclic loading (with a bond strength of less than 20 MPa after 10⁷ fatigue cycles).^[^ ^45^ ^]^

The DEJ, a 50–100 µm transitional zone, exemplifies nature's hierarchical mechanoadaptation strategy (Figure 2a).^[^ ^46^ ^]^ Its “zig‐zag” collagen/HAP microstructure reduces stress transfer efficiency compared to homogeneous ceramics through biomechanical impedance mismatch, crack deflection along collagen fibrils, and viscoelastic energy dissipation through sacrificial bonds.^[^ ^47^ ^]^ Synthetic materials, however, struggle to reproduce this phenomenon at submicron scales.^[^ ^48^ ^]^ Similarly, cementum demonstrates adaptive mineralization strategies. Acellular extrinsic fiber cementum provides rigid anchorage, while cellular mixed stratified cementum maintains remodeling capacity. This duality highlights the need for restoratives that can balance interfacial stability with biological adaptability.^[^ ^49^ ^]^

Drawing from these structural insights, the design of next‐generation dental restorative biomaterials should focus on several key aspects. First, it should consider the precise alignment of HAP to mimic the enamel's multi‐scale anisotropy. Second, it should establish dynamic stress dissipation mechanisms, such as sacrificial bonds and dentin‐activated microfluidic channels with a diameter of 10–50 µm. Third, bioactive interfaces with staggered modulus gradients must also integrate regulatory signals, such as encapsulated TGF‐β/BMPs, to recruit endogenous stem cells and guide odontoblast‐like differentiation, thereby mimicking the dentin's innate regenerative environment. Emerging evidence has linked deviations from these biological principles to clinical failures. For example, homogeneous composites lacking enamel‐like gradients have higher wear rates, and non‐porous dentin adhesives suffer from reduced fatigue life due to stress concentration.^[^ ^50^, ^51^ ^]^

As the field progresses, truly biomimetic restoratives must move beyond superficial imitation. They should embrace the fundamental mechanobiological principles that govern natural dental tissues. By harmonizing the mechanical hierarchy with bioactive signaling, such as the controlled release of Wnt agonists or DPSC‐homing peptides, next‐generation materials could actively promote tissue regeneration rather than merely replacing lost structures. Only through such a deep integration of materials science and biological insight can we develop restoratives that are capable of withstanding the relentless mechanochemical challenges of the oral cavity while promoting true tissue regeneration.

## 3 Design and Materials Considerations

**Figure 3:**
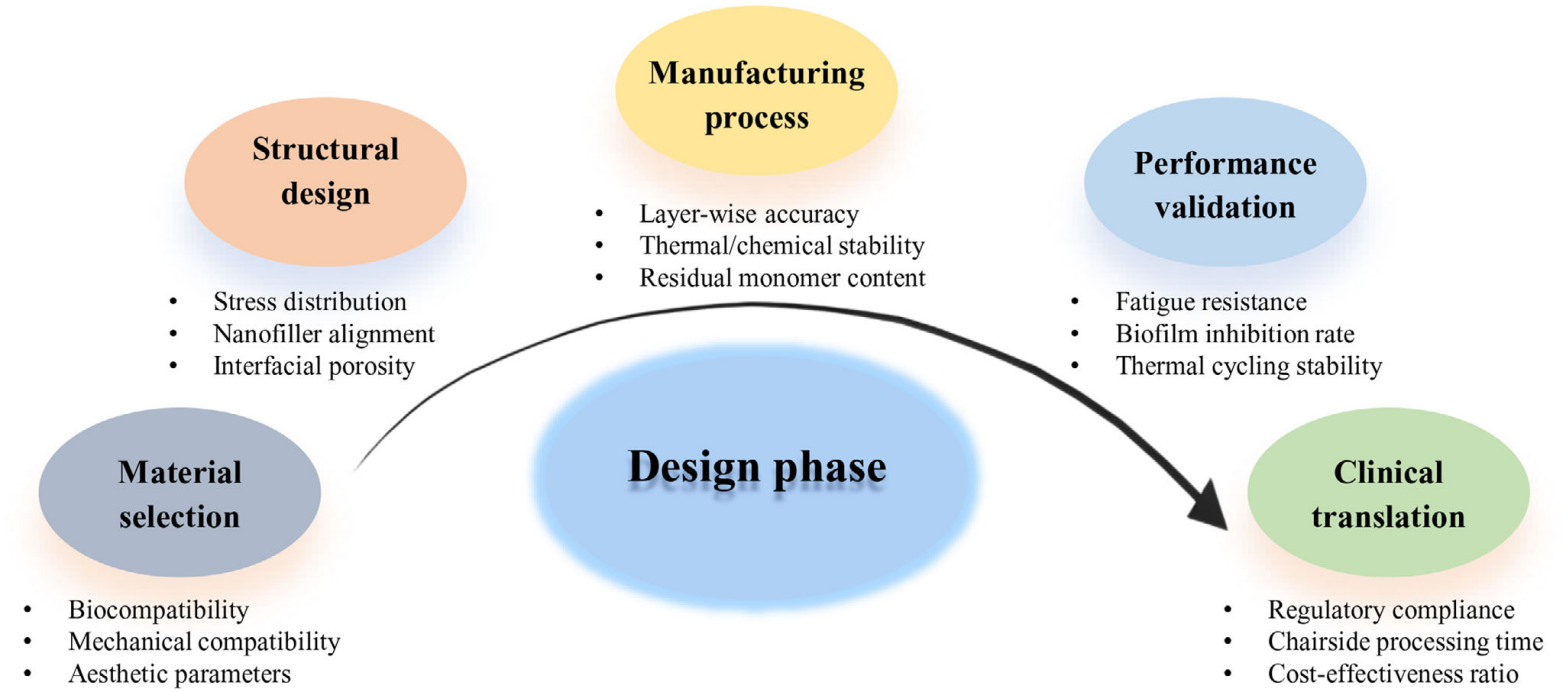
Pathway of materials design for dental defect restoration.

The next frontier in dental restorative materials lies in transcending mere structural replacement to achieve true biological integration. This requires a hierarchical design framework that simultaneously addresses multiscale mechanical compatibility (such as the enamel to dentin gradients), biointerfacial molecular signaling (e.g., adhesives inhibiting matrix metalloproteinases), and dynamic performance adaptation (pH/load‐responsive properties). Such paradigm shifts require the integration of computational materials design with mechanobiology principles. **Figure** **3** provides an overview of the design phases for dental defect repair materials, enumerating the primary considerations associated with each phase of the process.

Modern material selection strategies prioritize bioactive functionality while ensuring mechanical integrity. Bioactive glasses and calcium silicate cements facilitate interfacial remineralization through controlled ion release (Ca^2^⁺, PO_4_^3−^), but their inherent porosity impacts long‐term stability.^[^ ^52^, ^53^, ^54^, ^55^ ^]^ Hybrid systems that integrate zirconia frameworks with polymer‐infiltrated networks enhance stress distribution, and nanoscale engineering enables the simultaneous enhancement of fracture toughness and optical properties.^[^ ^56^, ^57^ ^]^ However, a crucial challenge is to prevent nanoparticle aggregation at functional concentrations without compromising aesthetic results.

**Figure 4:**
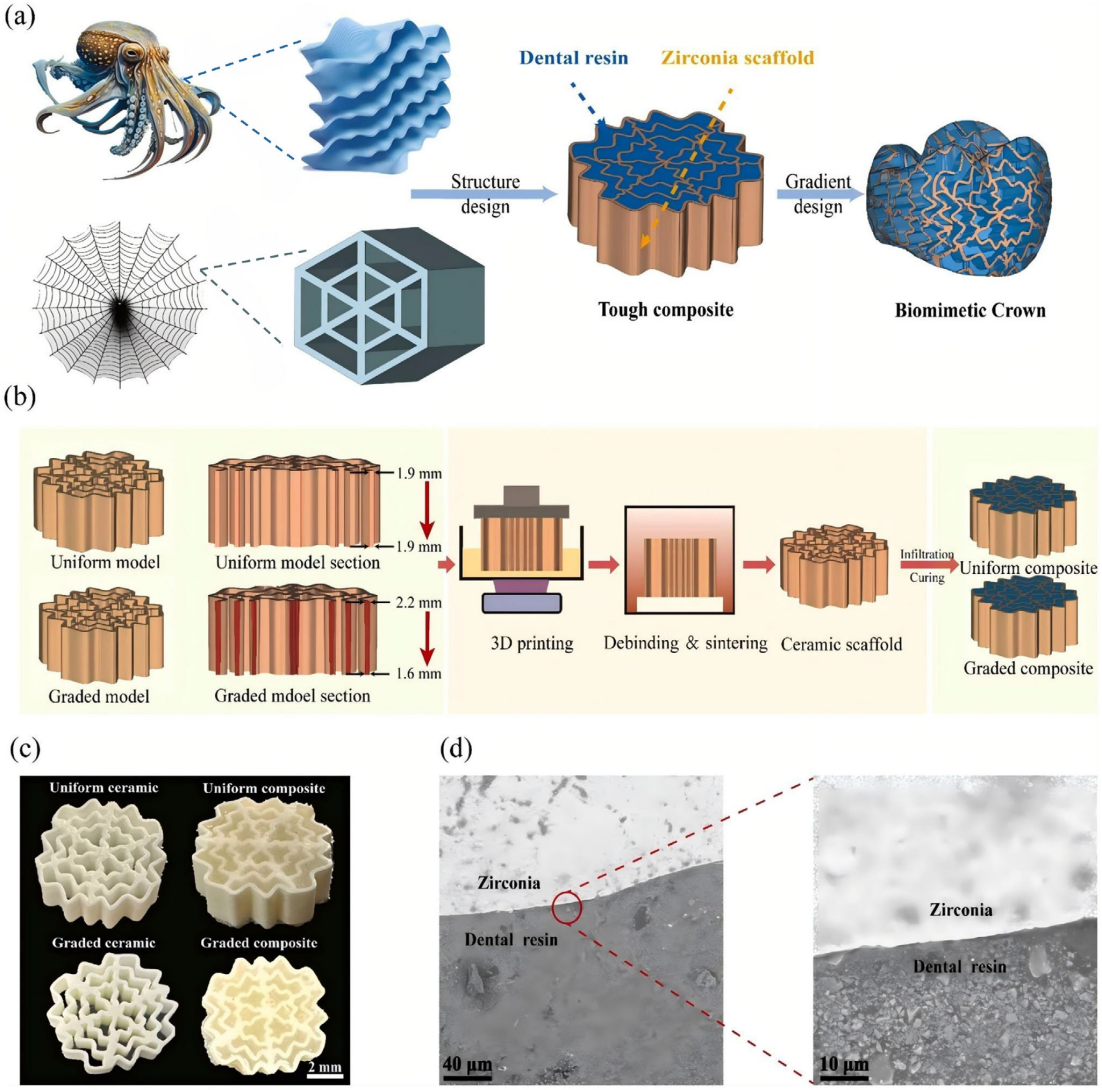
The design and fabrication schemes of the biomimetic gradient ceramic‐polymer composite crown. a) The integrated biomimetic strategy involves infiltrating S‐shaped hexagonal porous zirconia with resin to form the ceramic/polymer composite, featuring a gradient increase of ceramic wall thickness, ultimately constructing a biomimetic dental crown. b) Design and manufacturing process for the gradient/uniform porous ceramics with a volume fraction of 33.56 %. c) Photographs of the sintered gradient/uniform porous ceramic scaffold and cured composite material, with sample dimensions of 7.5 × 7.5 × 2.7 mm^3^. (d) SEM images of the ceramic‐polymer interface. Reproduced with permission.^[^ ^12^ ^]^ Copyright 2025, Elsevier.

Structural innovation focuses on biomimetic graded architectures that mimic the transitions found in natural tissues. The dentinoenamel junction (DEJ) employs a staggered arrangement of hydroxyapatite (HAP)‐collagen to deflect cracks and dissipate stress. Layer‐by‐layer additive manufacturing has now achieved modulus gradients (from 20 to 1 GPa over 200 µm) comparable to those of the DEJ, and advanced alignment techniques are approaching the anisotropic organization of natural enamel.^[^ ^58^, ^59^ ^]^ However, these advancements are offset by ongoing issues with residual stresses that jeopardize interfacial integrity, prompting research into novel stress‐absorbing interfacial designs. Functionally graded materials (FGMs) and polymer‐infiltrated ceramic networks (PICNs) are now capable of replicating the natural dentinoenamel modulus gradients. For instance, dental resin can infiltrate S‐shaped hexagonal porous zirconia structures to form a biomimetic crown composed of ceramic/polymer composites (**Figure** **4**a), thereby achieving a gradual increase in ceramic wall thickness.^[^ ^60^ ^]^ Nevertheless, the scalable fabrication of sub‐µm architectural features (similar to dentinal tubules) and fatigue‐resistant interfaces capable of withstanding cyclic loading requires further innovation.

Manufacturing technologies have revolutionized restorative fabrication. High‐resolution 3D printing techniques, such as digital light processing (DLP) and stereolithography, now achieve anatomical precision in crowns and bridges. This level of accuracy was previously unattainable.^[^ ^12^, ^61^ ^]^ Figure 4b presents the design and 3D printing fabrication process of the gradient porous ceramic, in which the controlled pore gradient contributes to optimized stress distribution and minimized residual stress at the interface. A significant advancement in the field is the integration of functional components within restoratives. Multi‐material printing systems now allow for the incorporation of conductive polymers and piezoelectric ceramics into dental restorations, enabling real‐time monitoring of occlusal forces and early detection of biomechanical overload, thus enhancing the functionality and safety of the restorations. However, practical constraints, particularly related to processing times and equipment requirements, hinder their widespread adoption. A key issue is achieving full densification of ceramics within clinically acceptable timeframes. For instance, while manufacturing technologies like 3D/4D printing and microwave sintering can enable precise geometries with ±25 µm accuracy and high‐density ceramics, scaling these processes for clinical chairside application remains challenging.^[^ ^62^ ^]^ Emerging solutions offer hope for overcoming these limitations. Microwave‐assisted sintering shows promise as it reduces processing times while minimizing grain growth. The sintered scaffold shown in Figure 4c highlights the clinical scalability of these processes, as its dimensions align with standard molar restoration requirements. Scanning electron microscopy (SEM) analysis in Figure 4d reveals the ceramic‐polymer interface quality, where minimal micro‐crack formation confirms the efficacy of microwave‐assisted sintering in maintaining structural integrity while reducing processing time. Additionally, two‐photon polymerization can be used for submicron resolution printing of bioactive scaffolds. Nevertheless, multi‐material integration, such as combining piezoelectric sensors with shape‐memory polymers, still faces challenges, including interfacial incompatibility and residual stress issues.

Functional integration is at the forefront of restorative innovation. Contemporary antimicrobial strategies have evolved from passive systems to smart, stimulus‐responsive release mechanisms, where bacterial proteases trigger the localized release of therapeutic agents such as chlorhexidine or lysozyme.^[^ ^63^ ^]^ Self‐healing materials incorporating microcapsules of triethylene glycol dimethacrylate (TEGDMA) or reversible Diels‐Alder adducts demonstrate crack closure efficiencies exceeding 80% under oral temperature cycling (5–55 °C), showcasing remarkable potential for extending restoration longevity. While bioactive interfaces aim to foster beneficial microbial ecosystems, phosphorylated polymer coatings selectively inhibit pathogenic bacteria while promoting commensal colonization.^[^ ^64^, ^65^ ^]^ These advancements necessitate equally advanced validation methodologies that better replicate the complex oral environment.

The future of dental restoratives is trending toward the development of truly adaptive materials that can respond to physiological changes. This vision encompasses 4D‐printed materials that react to environmental stimuli and personalized designs informed by real‐time biometric data, aiming for seamless integration with both hard and soft oral tissues. This could be achieved through innovations inspired by synthetic biology. By integrating data from electronic health records (EHRs) and intraoral sensors, personalized designs tailored to individual profiles can be realized.^[^ ^66^, ^67^ ^]^ The challenge lies in balancing biological dynamics with the responses of synthetic materials, ensuring compatibility and maintaining microbial balance.^[^ ^68^, ^69^ ^]^ Engineering biofilms through genetic circuits could open the door to self‐evolving, next‐generation materials.^[^ ^10^ ^]^

Dental restorative materials must undergo rigorous testing to ensure biocompatibility, mechanical stability, and functional performance before clinical application. Both in vitro (laboratory‐based) and in vivo (animal/clinical) models are essential for comprehensive evaluation (**Table** **1**). While traditional mechanical testing remains crucial, there is a growing emphasis on biologically relevant assessment models that incorporate microbial, biochemical, and biomechanical factors to better predict clinical performance and expedite the translation of innovative materials to patient care. In vitro methodologies quantify mechanical properties but often overlook environmental variables. Accelerated aging protocols, such as thermomechanical cycling, offer insights into long‐term performance but often fall short in replicating the enzymatic and abrasive complexities of the oral cavity.^[^ ^70^, ^71^ ^]^ Biofilm reactors better simulate oral microbial ecology, providing more clinically relevant assessments of antimicrobial efficacy and surface degradation, but lack host immune responses.^[^ ^72^, ^73^, ^74^, ^75^, ^76^ ^]^ In vivo models, such as rodent subdermal implants, assess inflammatory responses (e.g., IL‐6, TNF‐α levels) but poorly replicate the oral mechanical environment.^[^ ^77^ ^]^ Canine crown models provide closer approximations of human mastication but are constrained by ethical and logistical challenges.^[^ ^78^ ^]^ Human clinical trials remain the gold standard, tracking long‐term survival rates and secondary caries incidence, yet their high costs and ethical complexities necessitate robust preclinical validation, highlighting the need for surrogate endpoints, such as marginal adaptation or biofilm inhibition rates, to accelerate regulatory approval. A tiered testing strategy combining in vitro screening (cell/biomechanical assays) with in vivo validation (rodent→large animal→clinical trials) ensures robust evaluation of dental restorative materials. Future research should focus on advanced biomimetic models (e.g., human‐on‐chip microphysiological systems that simulate the oral microenvironment) to bridge the gap between lab findings and clinical outcomes. Additionally, the trend toward AI‐assisted prediction modeling can reduce animal testing.

**Table 1:** In vitro and in vivo models for the evaluation of dental defect restoration materials.

Model type		Mechanism	Refs.
In vitro			
Mechanical and physicochemical testing	Compressive and flexural strength	ISO 9917, ISO 4049	[11, 12, 15, 17, 19, 28, 29, 56, 70, 79, 80, 81, 82, 83, 84, 85, 86, 87, 88, 89, 90, 91, 92, 93, 94, 95, 96, 97, 98, 99, 100, 101]
	Wear resistance	Chewing simulators, 50–100k cycles	[11, 17, 71, 94, 96, 102]
	Degradation and ion release	pH cycling, aging in artificial saliva	[7, 103, 104, 105, 106, 107, 108]
Cell‐based assays	hDPSCs	Odontogenic differentiation (ALP, DSPP, DMP‐1 markers)	[109, 110, 111, 112, 113, 114, 115, 116]
	Gingival fibroblasts	Soft tissue integration and inflammatory response (TNF‐α, IL‐6)	[21, 117, 118]
	MC3T3‐E1 (osteoblasts)	Bone regeneration potential	[82, 119, 120]
	THP‐1 (macrophages)	Immunomodulatory effects (M1/M2 polarization)	[121]
Biofilm and antimicrobial models	Streptococcus mutans Porphyromonas gingivalis	Biofilms on material surfaces	[7, 9, 19, 72, 76, 107, 117, 122, 123, 124, 125, 126, 127, 128, 129]
	Live/dead staining CFU counting	Antibacterial efficacy	[107, 127, 130, 131]
3D bioprinted and organoid models	Dentin‐pulp complex organoids	Mimic natural tissue responses	[38, 132]
	Microfluidic chips	Simulate dynamic oral environment (pH, enzymes, shear forces)	[133, 134]
In Vivo			
Small animal models (rodents)	Rat molar defect model	Drill‐induced cavities test pulp capping/regeneration Micro‐CT analysis of mineralized tissue formation	[49, 135, 136]
Large animal models (pigs, dogs, non‐human primates)	Canine periodontal defect model	Ligature‐induced periodontitis test guided tissue regeneration membranes	[32, 71, 78, 113, 137, 138]
	Swine tooth extraction socket model	Alveolar bone preservation compares graft materials	[34, 102, 139, 140, 141]
Clinical trials (human studies)	Phase I/II trials	Safety & biocompatibility (ISO 10 993)	[62, 63, 125, 142, 143, 144, 145, 146, 147, 148, 149, 150, 151, 152]
	Phase III/IV trials	Long‐term success rates (5–10 year follow‐up)	[45, 60, 77, 125, 128, 153, 154]

## 4 Materials Innovation

The advancement of materials for dental defect restoration has undergone a profound transformation, shifting from traditional passive replacements to sophisticated bioactive and intelligent systems capable of dynamically interacting with the oral environment (**Table** **2**). This evolution is driven by the necessity to restore dental function and to actively promote oral health through integration with biological tissues and responsiveness to physiological changes.

**Table 2:** Overview of common material types for dental defect restoration.

Category		Key composition	Limitations	Clinical application	Refs.
Conventional dental restorative materials	Dental amalgam	Mercury (45–50 wt %), Ag‐Sn‐Cu alloy (γ‐phase)	Mercury toxicity, poor aesthetics	Posterior restorations (phased out)	[13, 151, 155, 156, 157]
	Gallium‐based alloys	Gallium, indium, tin	Low mechanical strength, high cost	Anterior teeth restoration	[158, 159]
	Zirconia (3Y/4Y/5Y‐TZP)	ZrO_2_ stabilized with Y_2_O_3_ (3 mol %)	Low‐temperature degradation, high cost	Crowns, bridges, implants	[95, 98, 100, 160]
	Resin‐based composites	Bis‐GMA/TEGDMA matrix, SiO_2_/ZrO_2_ fillers (60–80 vol %)	Polymerization shrinkage (1.5–2.5%), biofilm adhesion	Anterior/posterior restorations	[21, 85, 118, 161, 162]
	Glass ionomer cements	Alumino‐silicate glass, polyacrylic acid	Low wear resistance, moisture sensitivity	Class V cavities, luting agents, pediatric	[79, 163, 164, 165]
	Lithium disilicate	SiO_2_‐Li_2_O‐K_2_O glass‐ceramic	Brittle, limited thickness capacity	Veneers, inlays, anterior crowns	[37, 93]
Bioactive and biomimetic materials	Calcium silicate cements	Tricalcium silicate, dicalcium silicate	Slow setting (45–60 min), discoloration	Pulp capping, root repair	[53, 166, 167]
	Collagen‐hydroxyapatite composites	Collagen and hydroxyapatite	Mechanical strength was insufficient, and degradation rate matched poorly	Auxiliary restoration after dental caries filling, alveolar bone defect repair	[108, 114, 115, 168, 169]
	Bioactive glasses	SiO_2_‐CaO‐Na_2_O‐P_2_O_5_ (45S5 Bioglass)	Brittle, low mechanical strength	Periodontal repair, bone grafts	[106, 163, 170, 171]
	Decellularized extracellular matrix (dECM)	Collagen, elastin, glycosaminoglycans	Quality is difficult to control, and mechanical properties are insufficient	Periodontal tissue repair membrane	[142, 149, 172, 173]
Smart‐responsive materials	pH‐responsive hydrogels	Poly (ethylene glycol)‐poly (β‐amino ester)	Limited long‐term stability	Secondary caries prevention	[63, 174, 175, 176, 177]
	Shape‐memory polymers	Polyurethane/polycaprolactone blends	Low thermal resistance	Marginal seal enhancement	[67, 119, 178]
	Piezoelectric composites	BaTiO_3_/PMMA or resin matrix	Complex fabrication, cost	Occlusal force monitoring, antimicrobial	[179, 180, 181, 182, 183]
	Enzyme‐responsive biomaterials	Specific enzymes that can respond, natural or synthetic polymers	Susceptible enzyme activity, complex design and high cost	Treatment of endodontic and periodontal diseases	[8, 128, 184, 185]
	Light‐activated systems	Photoinitiator, monomer or prepolymer	Limited light depth	Tooth filling, bonding of orthodontic materials	[86, 186, 187, 188]

### 4.1 Conventional Materials

The foundation of modern dental restoration lies in conventional materials that have undergone iterative refinement over decades, achieving a delicate balance between mechanical performance, biocompatibility, and clinical practicality. These materials—metallic alloys, ceramics, resin‐based composites, and glass ionomer cements—remain cornerstones of restorative dentistry despite the emergence of advanced alternatives. Their enduring relevance stems from well‐characterized properties, standardized fabrication protocols, and extensive clinical validation, although each category faces unique challenges that have spurred incremental innovations.

Metallic alloys, particularly dental amalgams, once dominated restorative dentistry due to their exceptional compressive strength (300–400 MPa) and wear resistance. However, concerns over mercury toxicity have driven the development of gallium‐based alternatives, though their clinical performance remains inferior due to oxidation issues.^[^ ^151^, ^155^, ^158^, ^189^, ^190^ ^]^ Dental ceramics represent a significant advancement, with 3Y‐TZP zirconia (3 mol % yttria‐stabilized tetragonal zirconia polycrystal) offering outstanding flexural strength (900–1200 MPa) through transformation toughening mechanisms.^[^ ^191^ ^]^ The introduction of high‐translucency 5Y‐TZP formulations addresses aesthetic demands but compromises mechanical properties.^[^ ^92^ ^]^ Lithium disilicate glass‐ceramics have become the gold standard for anterior restorations, combining excellent fracture toughness with unparalleled optical properties that mimic natural dentition.^[^ ^93^, ^192^ ^]^

Resin‐based composites revolutionized minimally invasive dentistry through their direct placement capability and tooth‐colored aesthetics.^[^ ^95^, ^193^, ^194^, ^195^ ^]^ Modern formulations incorporate 60–80 vol % nanofillers to optimize mechanical properties and surface smoothness. Bioactive composites integrate calcium phosphate or bioactive glass fillers (10–20 wt %), releasing ions (Ca^2^⁺, PO_4_^3−^, F^−^) to promote remineralization at the restoration margin.^[^ ^90^, ^196^ ^]^ However, polymerization shrinkage‐induced stresses continue to challenge marginal integrity, prompting innovations like bulk‐fill composites and alternative polymerization chemistries. Glass ionomer cements occupy a unique niche with their self‐adhesive properties and sustained fluoride release, though their mechanical limitations restrict use to low‐stress applications.^[^ ^79^, ^164^, ^165^, ^197^ ^]^

### 4.2 Bioactive and Biomimetic Materials

Bioactive and biomimetic materials signify a transformative shift in dental restoration, focusing on establishing stable interactions with biological tissues and mimicking the structural and functional hierarchy of natural dental tissues over time. These materials are engineered to promote biomineralization, antimicrobial activity, and tissue integration through their inherent properties and compositions. Furthermore, the material system for dental defect repair also incorporates profuse cells and bioactive molecules, as outlined in **Table** **3**. These components include DPSCs that differentiate into odontoblasts, BMP‐2 and Wnt signaling molecules enhancing biomineralization/stem cell proliferation, and adhesive proteins have the potential to induce tooth regeneration and facilitate sustained integration between the teeth and the repair materials, thereby emerging as a focal area of research.

**Table 3:** Biological components of dental restorative biomaterial systems.

Category	Primary function	Clinical applications	Refs.
*Cells*			
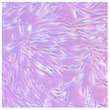 DPSCs	Self‐renewal, differentiation into odontoblasts	Pulp regeneration, dentin repair	[113, 115]
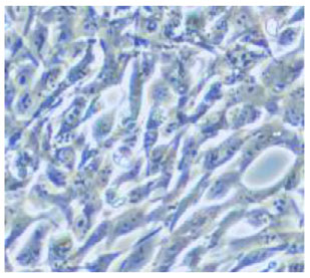 PDLSCs	Regenerate periodontal tissues, promote bone formation	Periodontal defect repair, tooth regeneration	[116, 143, 144]
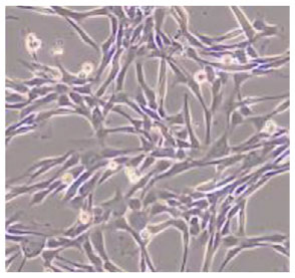 MSCs	Immunomodulation, tissue regeneration	Bone grafting, alveolar ridge augmentation	[113, 116, 141, 171, 198, 199]
*Growth factors*			
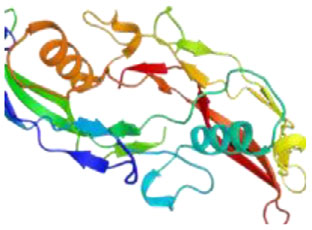 BMPs	Induce osteogenic/odontogenic differentiation	Bone regeneration, dentin‐pulp complex repair	[141, 200]
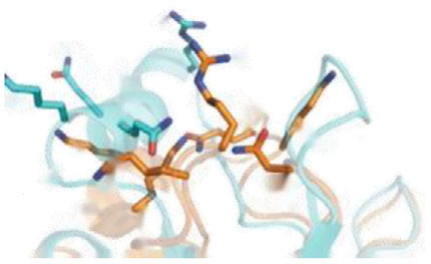 FGF	Promote angiogenesis, cell proliferation	Periodontal ligament repair, pulp revascularization	[115, 149]
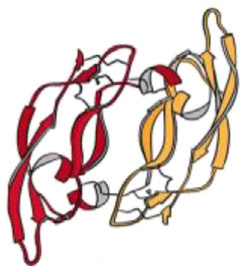 VEGF	Enhance vascularization, tissue remodeling	Bone grafts, implant osseointegration	[149, 170, 201]
*ECM*			
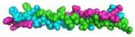 COL‐I	Structural scaffold, mineralization template	Dentin‐pulp scaffolds, guided bone repair	[202, 203]
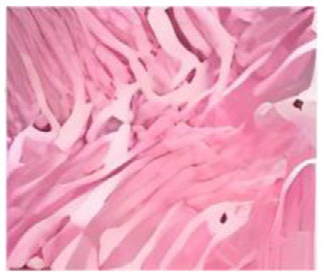 DDM^a)^	Preserve native ECM architecture, bioactive cues	Dentin regeneration, alveolar bone repair	[132, 149, 204]
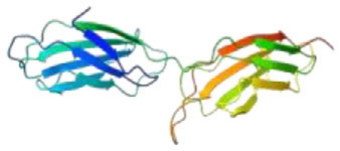 Fibronectin	Cell adhesion, migration, and differentiation	Bioactive coatings for implants	[205]
*Signaling molecules*			
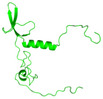 Wnt proteins	Regulate stem cell fate, promote tissue repair	Pulp regeneration, periodontal healing	[139, 141, 206]
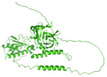 Hedgehog proteins	Modulate odontoblast differentiation	Dentin repair, tooth development models	[136, 207]
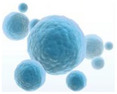 Exosomes	Paracrine signaling, anti‐inflammatory	Pulp revascularization, periodontal repair	[135, 173]
*Biomaterials*			
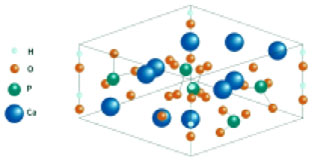 Bioceramic (e.g., hydroxyapatite, 45S5)	Stimulate hydroxyapatite formation, antimicrobial	Bone grafts, enamel remineralization	[56, 106, 170, 171]
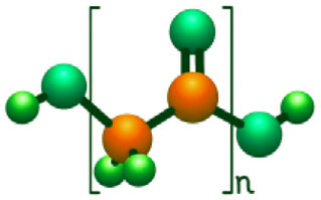 Synthetic polymer (e.g., PLA^b)^, PGA^c)^)	Serve as scaffolds or carriers for cells and bioactive molecules	Scaffolds, root canal posts	[154, 208, 209]
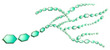 Natural polymers (e.g., alginate, chitosan)	Antimicrobial, mucoadhesive, hydrogel carrier, 3D bioprinting	Bioactive liners, drug delivery systems, pulp encapsulation, periodontal scaffolds	[141, 154, 175, 208, 210, 211]
*Cytokines*			
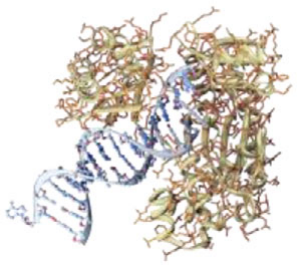 TGF‐β	Immunomodulation, fibrosis regulation	Pulp capping, periodontal tissue repair	[149, 170]
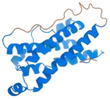 IL‐6	Pro‐inflammatory/anti‐inflammatory dual role	Wound healing, infection control	[91, 122, 212]
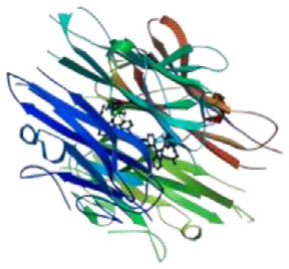 TNF‐α	Regulate immune response, tissue remodeling	Peri‐implantitis therapy	[122, 212, 213]
*Adhesive proteins*			
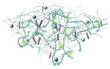 Laminin	Cell adhesion, basement membrane formation	Dental epithelial regeneration, implants	[154]
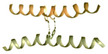 Vitronectin	Promote cell attachment, stabilize ECM	Bioactive coatings, tissue‐engineered constructs	[214]
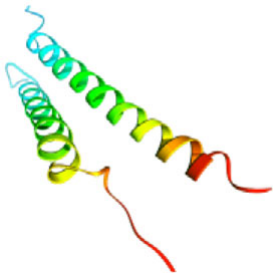 Integrins	Promotes cell adhesion and signaling	Bone graft materials, biological scaffolds	[49, 215]

^a)^ Decellularized dentin matrix;

^b)^ Polylactic acid;

^c)^ Polyglycolic acid.

**Figure 5:**
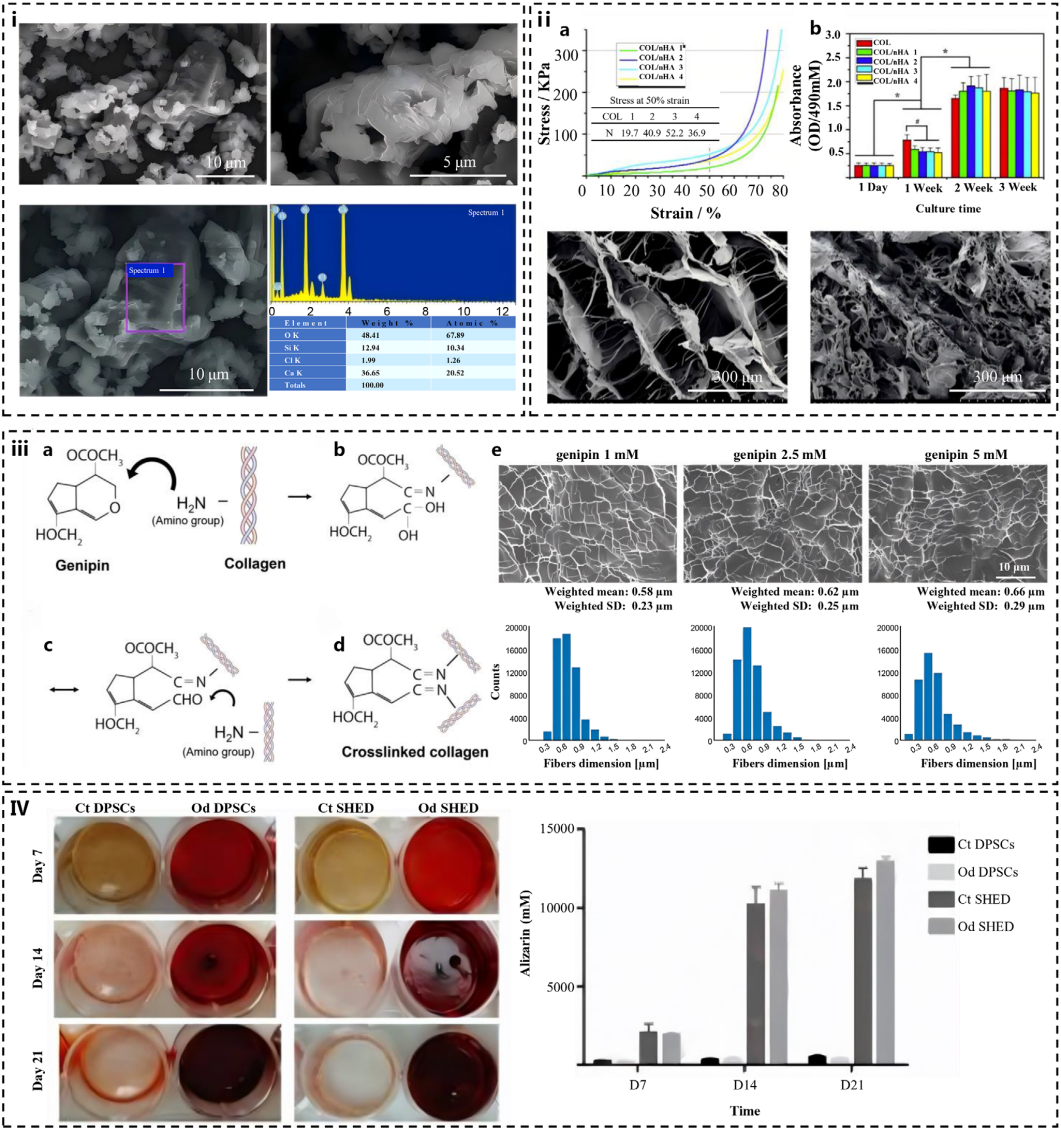
Research results of bioactive dental materials. (i) SEM and EDS analysis results of Calcium Silicate‐Based Endodontic Cements. Reproduced with permission.^[^ ^166^ ^]^ Copyright 2018, Wiley. (ii) Compression testing and MTT results of collagen/nano‐hydroxyapatite scaffolds with surface morphology comparisons. (a) Compression performance curves and stress values of scaffolds at 50% strain. (b) MTT values of scaffolds in 3‐week culture. (c) Surface morphology of COL/nHA 2 scaffold after 1‐day culture. (d) Surface morphology of COL/nHA 2 scaffold after 3‐weeks culture. Reproduced with permission.^[^ ^82^ ^]^ Copyright 2016, Wiley. (iii) Schematic representation of the crosslinking mechanism of genipin. (a) Genipin interaction with primary amine groups. (b) Ring‐opening reaction in genipin and covalent bond with the amino group of collagen. (c) Formation of an unstable intermediate aldehyde group. (d) Formation of a new covalent bond with another polimer, which leads to the formation of the crosslink. (e) SEM images of JellaGel hydrogels crosslinked with genipin at different concentrations. They show the internal gel microarchitecture with the domains created by the collagen network. Scale bars = 20 µm. Reproduced under the terms of the CC BY 4.0 license. Permission obtained from the MDPI, Basel, Switzerland.^[^ ^168^ ^]^ Copyright 2021, Laura Riacci et al. (IV) ALP activity in DPSCs and SHED (stem cells from human exfoliated deciduous teeth). Following cultivation in odontogenic (Od) differentiation medium for 7, 14, and 21 days, cells were characterized in terms of their ALP activity. Cells cultivated in non‐odontogenic differentiation medium were used as controls. Reproduced with permission.^[^ ^113^ ^]^ Copyright 2020, Elsevier.

Calcium silicate cements exemplify the recreation of a dynamic interface between synthetic materials and biological tissues, promoting hydroxyapatite nucleation at the material‐dentin interface. SEM revealed the presence of relatively large particles coated by smaller ones, and energy‐dispersive X‐ray spectroscopy (EDS) confirmed that these particles are predominantly composed of calcium and silicon (**Figure** **5**i). The alkaline environment generated during their setting not only facilitates mineral deposition but also provides inherent antimicrobial activity against endodontic pathogens. Mineral trioxide aggregate, primarily composed of tricalcium silicate, dicalcium silicate, and bismuth oxide, hydrates to form calcium silicate hydrate gel and portlandite, creating an alkaline environment (pH 10–12) that promotes hydroxyapatite nucleation at the material‐dentin interface.^[^ ^131^, ^166^, ^167^ ^]^ This bioactivity allows mineral trioxide aggregate to seal marginal gaps through apatite deposition, achieving sealing efficiencies of 85–95% in root canal therapies. Biodentine, a faster‐setting variant, incorporates zirconia as a radiopacifier and calcium carbonate to accelerate hydration, reducing setting times to 12–15 min. Despite their efficacy, these cements face challenges such as tooth discoloration due to bismuth oxide oxidation and low flexural strength (8–12 MPa), restricting their use to non‐load‐bearing applications.^[^ ^166^ ^]^ Recent advances include doping with strontium or zinc ions to enhance antimicrobial activity (achieving a 3–4 log reduction against Enterococcus faecalis) and substituting bismuth with tantalum oxide to prevent discoloration.^[^ ^130^, ^131^, ^216^ ^]^

Collagen‐hydroxyapatite composites directly emulate dentin's organic‐inorganic architecture, combining type I collagen fibrils (50–100 nm diameter) with nano‐hydroxyapatite (nHA) particles (20–50 nm). Figure 5ii(a–d) shows that the compressive strength of these materials exhibited a notable enhancement, and the stress values at 50% strain demonstrated a correlation with the nHA content, where the optimal mass ratio was determined to be 1:3. They support cellular activity and odontogenic differentiation while providing mechanical properties suitable for load‐bearing applications. The collagen matrix provides viscoelasticity and serves as a scaffold for cell adhesion, while nHA replicates dentin's mineral phase, achieving compressive strengths of 80–120 MPa.^[^ ^82^, ^198^ ^]^ Crosslinking agents like riboflavin (under UV light) or genipin (a natural iridoid) are employed to stabilize the collagen network, as shown in Figure 5iii(a–d), where genipin's covalent bonding mechanism (ring‐opening reaction) enhanced resistance to collagenase degradation by 3.2 fold (Figure 5iii(e)).^[^ ^168^, ^169^ ^]^ These composites demonstrate exceptional biocompatibility, supporting odontoblast proliferation and differentiation in vitro, with gene expression markers (DSPP, DMP‐1) upregulated by 2–3 fold compared to traditional resin composites.^[^ ^108^, ^217^ ^]^ However, their susceptibility to hydrolytic degradation in saliva necessitates protective coatings such as peptide layers or silica infiltration.

Recent advances in enamel biomimetic restorative materials provide promising strategies for replicating the hierarchical structure and function of natural enamel. For example, amelogenin‐derived peptides have been shown to guide in vitro remineralization, forming a continuous hydroxyapatite layer with improved hardness and elastic modulus, closely resembling native enamel.^[^ ^218^ ^]^ Similarly, DNA‐guided hydroxyapatite composites engineered via biomineralization scaffolds have demonstrated dentin‐like stiffness (≈25 GPa), high tensile strength, machinability for CAD/CAM processing, and long‐lasting antibacterial activity, enabling their application in customized dental inlays.^[^ ^219^ ^]^ Parallel to this, an innovative RNA‐ACP/RNase system mimics the natural enamel maturation process. By leveraging RNA degradation to induce organic residue removal, this strategy facilitates lateral crystal growth, ultimately producing dense apatite crystals devoid of organic interference. The resulting enamel restorations demonstrate significantly enhanced mechanical performance and mineralization levels, addressing a critical limitation in conventional repair methods.^[^ ^220^ ^]^ In addition, nanosized calcium‐deficient hydroxyapatites, including Sr‐ and F‐substituted variants, have been shown to deposit enamel‐like CaP layers of ≈5–6 µm within one week of daily application, providing protective and remineralizing effects against erosion and caries.^[^ ^221^ ^]^ More recently, biomimetic scaffolds such as phosphorylated nano‐chitosan with amorphous calcium phosphate (Pchi‐ACP) and self‐assembling peptide P11‐4 have demonstrated strong remineralizing potential for early enamel white spot lesions, with Pchi‐ACP achieving up to a 63% reduction in lesion depth and formation of smooth, mineral‐rich enamel surfaces.^[^ ^222^ ^]^ Collectively, these biomimetic approaches illustrate how molecular design and nanotechnology can be harnessed to restore both the mechanical performance and biological resilience of natural enamel.

Bioactive glasses, particularly the 45S5 (composition: 45% SiO_2_, 24.5% CaO, 24.5% Na_2_O, 6% P_2_O_5_), extend the biomimetic concept further through controlled ion release that stimulates both mineralization and antibacterial effects. Upon contact with physiological fluids, these glasses undergo ion exchange, releasing Ca^2^⁺, Na⁺, and PO_4_^3−^ ions, which nucleate hydroxycarbonate apatite layers that chemically bond to dentin. The alkaline pH generated during dissolution (up to pH 8.5) further inhibits bacterial growth, reducing Streptococcus mutans biofilm formation.^[^ ^106^, ^170^ ^]^ To overcome inherent brittleness, hybrid systems like glass‐polymer composites have been developed, achieving flexural strengths while retaining bioactivity.^[^ ^84^ ^]^ Emerging formulations incorporate therapeutic ions (e.g., boron for angiogenesis, silver for antimicrobial action), enabling multifunctional responses tailored to clinical needs.^[^ ^104^, ^223^, ^224^ ^]^

Decellularized Extracellular Matrix (dECM) scaffolds represent a biomimetic leap, preserving the native architecture and signaling molecules of dental tissues.^[^ ^147^, ^172^ ^]^ Dentin‐derived dECM, processed via freeze‐thaw cycles and detergent treatments, retains collagen fibrils, glycosaminoglycans, and growth factors (BMP‐2, TGF‐β).^[^ ^149^, ^173^ ^]^ When combined with DPSCs, these scaffolds demonstrate remarkable potential for regenerative applications, promoting tissue formation through both structural and biochemical cues. These scaffolds promote odontogenic differentiation, with ALP activity 2.5 times higher than synthetic scaffolds (Figure 5IV) when seeded with dental pulp stem cells.^[^ ^113^ ^]^ Clinical trials using dECM for alveolar ridge augmentation demonstrate 30–40% greater bone volume retention compared to autografts, attributed to the scaffold's ability to recruit endogenous stem cells.^[^ ^142^ ^]^ Challenges include batch variability and immune responses to residual xenogenic antigens, driving research toward recombinant human ECM proteins (e.g., recombinant type I collagen) synthesized via yeast or bacterial expression systems.^[^ ^225^, ^226^ ^]^ However, challenges remain in standardizing these biologically derived materials and ensuring consistent clinical performance.

While bioactive materials offer unprecedented biological integration, their clinical translation is hindered by mechanical‐bioactivity trade‐offs, long‐term stability issues, and manufacturing complexity. Innovations such as nanocomposite designs, hermetic coatings, and advanced fabrication techniques (e.g., melt electrowriting or two‐photon polymerization) are addressing these challenges, paving the way for the next generation of bioactive and biomimetic materials. These advanced fabrication techniques enable the creation of multiscale biomimetic structures (e.g., dentinal tubule‐like microchannels), although they remain cost‐prohibitive for routine use. In summary, bioactive and biomimetic materials have shown great potential in dental restoration by promoting biological integration and mimicking natural tissue functions. However, the oral cavity is a highly dynamic and complex environment, which poses new challenges for these materials.

### 4.3 Smart‐Responsive Materials

**Figure 6:**
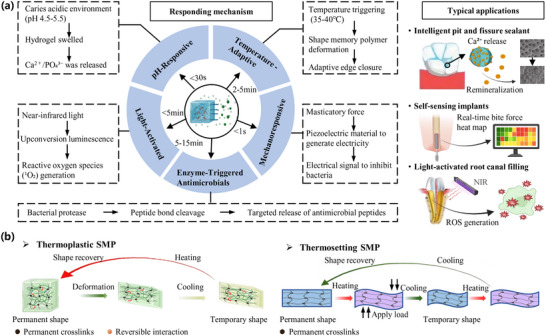
Infographic of smart‐responsive mechanism. a) The mechanism types of shape memory polymer (SMP) materials involved in dental defect repair materials. NIR: near‐infrared rays; ROS: reactive oxygen species. b) Schematic of the shape memory mechanism for thermoplastic SMP and thermosetting SMP.

To address the challenges posed by the dynamic oral microenvironment, smart‐responsive materials have emerged as a promising solution. These materials leverage stimuli‐responsive mechanisms—triggered by pH shifts, temperature fluctuations, mechanical stress, enzymatic activity, or light—to deliver targeted therapeutic actions, self‐repair microdamage, or optimize biomechanical performance in real time (**Figure** **6**a).^[^ ^63^, ^67^, ^119^, ^174^, ^175^, ^176^, ^177^, ^226^ ^]^ These materials hold great potential for addressing long‐standing challenges such as secondary caries, interface delamination, and biofilm accumulation.

pH‐responsive formulations exploit the acidic microenvironment (pH 4.5–5.5) of cariogenic biofilms. They trigger antimicrobial or remineralizing actions, offering targeted therapy while minimizing disruption to the oral microbiome.^[^ ^107^ ^]^ Poly(ethylene glycol)‐poly(β‐amino ester) hydrogels undergo protonation in low‐pH conditions, swelling to release preloaded agents such as chlorhexidine or amorphous calcium phosphate (ACP) nanoparticles.^[^ ^227^ ^]^ In vitro studies demonstrate a 60–70% reduction in Streptococcus mutans biofilm biomass under acidic challenge, coupled with ACP‐driven enamel remineralization.^[^ ^125^ ^]^ Dual pH‐responsive systems, such as layered mesoporous silica nanoparticles, sequentially release antibacterial agents at pH <5.5 and calcium ions at neutral pH, achieving synergistic caries inhibition and mineral repair.^[^ ^7^ ^]^ However, long‐term stability remains a concern, as repeated pH cycling degrades polymer matrices, reducing agent release efficiency by 30–40% after 6 months. Innovations like covalent adaptable networks with reversible imine bonds offer self‐healing capabilities, restoring pH responsiveness after acid‐induced damage.^[^ ^228^ ^]^

Temperature‐adaptive polymers offer innovative solutions for improving restoration adaptation and longevity. These materials harness the subtle thermal variations in the oral cavity (25–37 °C) or clinician‐controlled stimuli to optimize restoration performance. Shape‐memory polyurethanes with glass transition temperatures (Tg) near 35 °C enable self‐fitting restorations: as illustrated in Figure 6b, thermoplastic SMPs (left) adapt their shape through the mobility of molecular chain segments when heated above Tg, and the new shape is fixed upon cooling. In contrast, thermosetting SMPs (right) depend on permanent cross‐linking networks, enabling shape recovery through elastic deformation upon heating, without the occurrence of chain flow.^[^ ^229^, ^230^ ^]^ Trials report 40–50% lower microleakage in shape‐memory polyurethane‐based composites compared to conventional resins, with 90% shape recovery after thermocycling (5–55 °C, 10⁴ cycles).^[^ ^231^ ^]^ Thermo‐responsive hydrogels like poly(N‐isopropylacrylamide) transition from hydrophilic to hydrophobic states above 32 °C, enabling on‐demand drug release during inflammatory fever episodes.^[^ ^221^ ^]^ For instance, poly(N‐isopropylacrylamide) loaded with dexamethasone reduces inflammation markers (TNF‐α) in retinal‐pigmented epithelial cell line hTERT models when triggered by localized temperature increases.^[^ ^140^ ^]^ Challenges include unintended activation during hot food/drink consumption and fatigue resistance under cyclic thermal loads, necessitating precise Tg tuning and crosslinker optimization.

Mechanoresponsive composites represent another frontier, converting occlusal forces into beneficial biological effects.^[^ ^232^ ^]^ Piezoelectric materials generate electrical potentials under masticatory loading that disrupt bacterial adhesion. Barium titanate (BaTiO_3_)‐reinforced resins generate surface potentials (50–200 mV) under masticatory loads, which disrupt bacterial membrane potentials via electrostatic interactions, achieving inhibition of S. mutans adhesion at 1–2 Hz loading frequencies.^[^ ^126^ ^]^ Triboelectric systems harness chewing energy to power integrated sensors for real‐time monitoring. Triboelectric nanogenerators embedded in restorations harness friction energy from chewing to power integrated sensors, enabling real‐time monitoring of occlusal forces and early detection of bruxism.^[^ ^153^ ^]^ Despite their promise, piezoelectric materials face challenges in poling alignment during manufacturing, as misoriented BaTiO_3_ crystals reduce output voltages. Additive manufacturing techniques like direct ink writing with in situ electric field alignment may address this.^[^ ^233^ ^]^

Enzyme‐activated biomaterials provide exquisite biological specificity, responding to pathological proteases while ignoring normal physiological conditions. Matrix metalloproteinases (MMPs)‐sensitive peptide‐conjugated hydrogels degrade specifically in the presence of inflammatory proteases, releasing encapsulated antimicrobials (e.g., chlorhexidine) or growth factors (BMP‐7) at disease sites.^[^ ^184^ ^]^ In periodontal defect models, these hydrogels reduce inflammation markers (IL‐1β, TNF‐α) while enhancing bone regeneration. Similarly, lysozyme‐triggered systems exploit the enzyme's prevalence in cariogenic biofilms, activating dormant antibacterial agents like quaternary ammonium compounds.^[^ ^8^ ^]^ Enzyme‐triggered antimicrobials target pathogen‐specific proteases to minimize off‐target effects on commensal flora. Peptide‐conjugated hydrogels incorporating MMP‐9 cleavable linkers release antimicrobial peptides like LL‐37 selectively in inflamed or infected regions. In periodontal defect models, these systems reduce Porphyromonas gingivalis counts by 3–4 log units while sparing beneficial Streptococcus salivarius.^[^ ^128^ ^]^ Similarly, lysozyme‐responsive nanoparticles release hydrogen peroxide (H_2_O_2_) upon contact with bacterial cell wall hydrolases, achieving 99% kill rates against Lactobacillus acidophilus within 30 min. To prevent premature activation, encapsulation strategies like layer‐by‐layer polyelectrolyte coatings ensure <5% drug leakage under physiological conditions, with full release triggered only by pathogenic enzymes.^[^ ^180^ ^]^ This approach enables precise targeting of inflammatory or infectious processes, potentially revolutionizing the management of periodontal disease and peri‐implantitis.

Light‐activated technologies offer spatiotemporal control over therapeutic actions, allowing clinicians to precisely direct antibacterial or regenerative effects. Upconversion nanoparticles (UCNPs) doped with Er^3^⁺/Yb^3^⁺ convert near‐infrared (NIR, 980 nm) light to visible wavelengths, activating titanium dioxide (TiO_2_) photocatalysts embedded in restorations.^[^ ^186^ ^]^ This generates reactive oxygen species that eradicate biofilms without thermal damage to surrounding tissues. NIR penetration depths of 3–5 mm enable biofilm eradication beneath composite margins or within dentinal tubules.^[^ ^186^, ^187^, ^188^, ^234^, ^235^ ^]^ Photodynamic therapy systems like indocyanine green (ICG) loaded resins achieve similar efficacy, with singlet oxygen (^1^O_2_) production rates of 0.5–1.0 µM min^−1^ under 808 nm irradiation.^[^ ^236^, ^237^ ^]^ Light‐triggered self‐healing materials, such as microcapsules containing TEGDMA and photoinitiators, achieve 80–90% crack closure under UV exposure, restoring flexural strength to 95% of original values.^[^ ^86^ ^]^

Clinical translation of these advanced materials faces several challenges. Mechanical property trade‐offs remain a persistent issue, particularly for bioactive systems where high ion release often correlates with reduced strength. Long‐term stability in the harsh oral environment presents another hurdle, as cyclic loading, temperature fluctuations, and enzymatic activity can degrade material performance over time. Strategies to mitigate these issues include using enzyme‐resistant monomers like urethane dimethacrylate and incorporating fatigue‐damping additives such as carbon nanotubes.^[^ ^87^, ^238^, ^239^ ^]^ Biocompatibility trade‐offs arise from nanoparticle leaching, which can induce cytotoxicity; surface functionalization with PEG or polydopamine can reduce leaching by 60–70%.^[^ ^240^, ^241^ ^]^ Manufacturing complexity and cost considerations further complicate widespread adoption, particularly for technologies requiring specialized equipment or processing conditions. Hybrid manufacturing approaches, such as 3D‐printed scaffolds with post‐printed bioactive coatings, help balance performance and scalability.^[^ ^242^ ^]^ The future trajectory of dental restorative materials points toward increasingly sophisticated biointegration. Next‐generation systems will likely incorporate elements of synthetic biology, creating materials that not only respond to their environment but also actively participate in biological signaling networks, enabling personalized and adaptive oral healthcare solutions.

## 5 Advanced Manufacturing and Performance Optimization

While functional responsiveness addresses clinical dynamics, manufacturing advances are equally critical to translate these innovations into practice. These advancements have granted unprecedented control over material architecture, composition, and functionality, enabling the creation of dental restoratives with enhanced performance and tailored properties. Modern manufacturing techniques have transcended the limitations of traditional methods by integrating bioinspired designs, achieving multiscale structural control, and incorporating dynamic responsiveness to the oral environment. This section delves into how these cutting‐edge technologies effectively bridge the gap between material science and biological outcomes, with a particular emphasis on the material‐structure‐cell interactions that are pivotal for the success of dental restorations.

### 5.1 Precision and Personalized Additive Manufacturing

**Figure 7:**
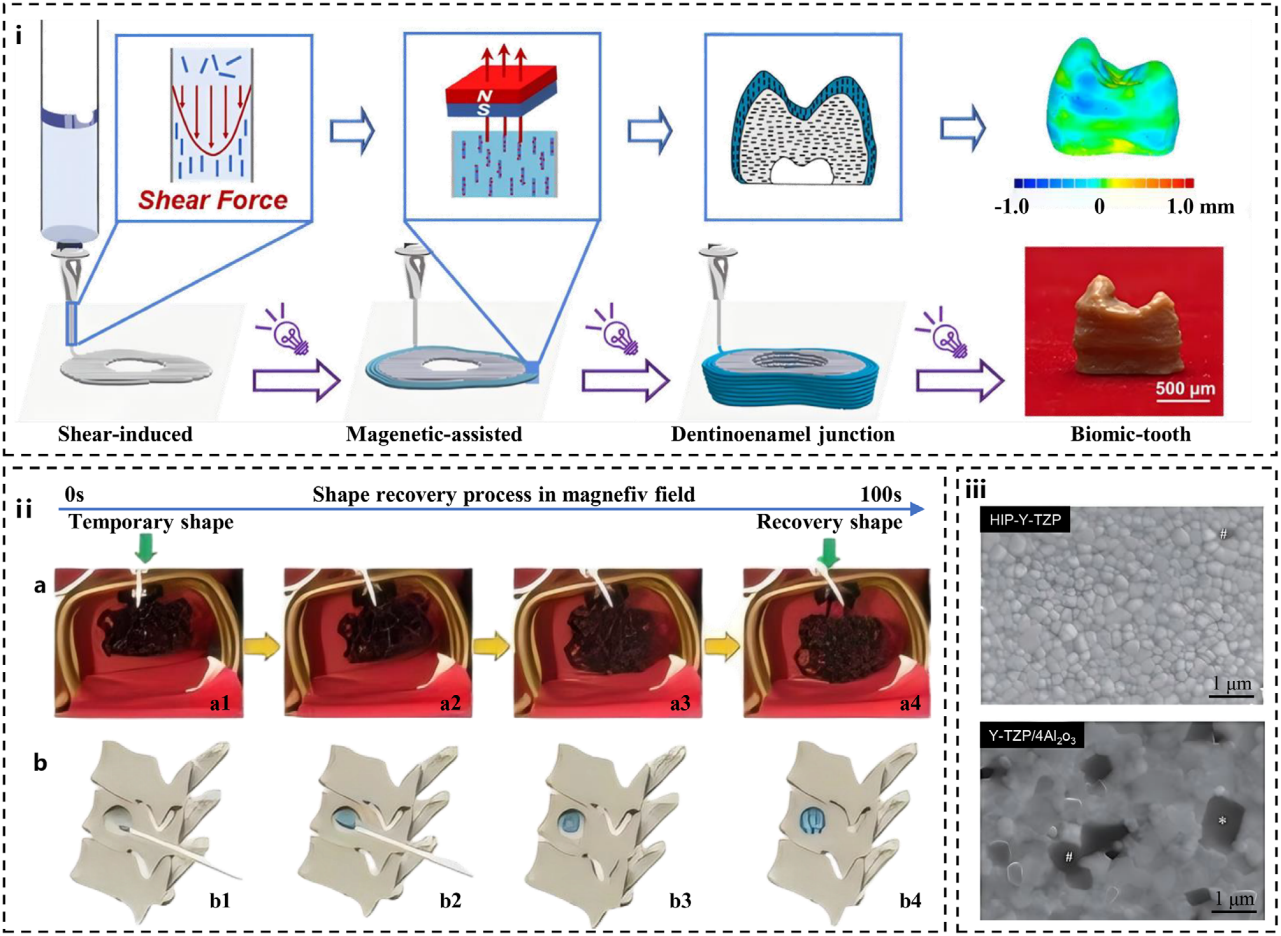
Application of additive manufacturing technology in the preparation of dental restoration materials. (i) 3D printing schematic diagram of biological tooth with multiple ordered hierarchical structures. Reproduced with permission.^[^ ^243^ ^]^ Copyright 2025, Elsevier. (ii) a) Shape recovery behavior of 4D‐printed stent in magnetic field and b) simulation of the mechanism of 4D‐printed stent. Reproduced with permission.^[^ ^67^ ^]^ Copyright 2019, Elsevier. (iii) Representative SEM images of as‐polished high‐strength Y‐TZP with thermal etching. #: micro defect, *: alumina particle. Reproduced with permission.^[^ ^89^ ^]^ Copyright 2020, JSDMD.

Additive manufacturing (AM) has revolutionized the field of dental restoration, primarily due to its unparalleled ability to produce patient‐specific, anatomically precise structures that surpass the constraints of conventional subtractive methods. The core advantage of AM lies in its capacity to fabricate complex geometries with micron‐scale resolution, which is indispensable for a wide range of applications, from customized prostheses to the recreation of biomic‐tooth with multiple ordered hierarchical structures.^[^ ^67^, ^243^, ^244^ ^]^ **Figure** **7**i illustrates the 3D printing process of biomic‐tooth.

Vat photopolymerization techniques, particularly DLP and stereolithography, have emerged as frontrunners in dental AM due to their exceptional precision and material versatility. These systems utilize UV or visible light to cure photopolymer resins layer‐by‐layer, achieving marginal accuracies of±25 µm. High‐performance resins, such as zirconia‐filled hybrids with 60–80 vol % ceramic loading, deliver flexural strengths of 120–150 MPa, suitable for long‐span prostheses like implant‐supported bridges.^[^ ^56^, ^71^, ^92^, ^95^, ^100^ ^]^ Recent advancements in resin chemistry, such as the development of UDMA formulations and reduced‐shrinkage monomers, have effectively addressed issues related to polymerization‐induced shrinkage and cytotoxicity. This has expanded the use of these resins to applications like direct pulp capping and pediatric dentistry.^[^ ^9^, ^95^, ^195^, ^245^ ^]^

Two‐photon polymerization, a specialized subset of vat photopolymerization, pushes the boundaries of resolution even further, enabling the production of submicron features (0.5–1.0 µm) that accurately replicate the prismatic microstructure of enamel. This capability is of utmost importance for the development of biomimetic surfaces that can significantly reduce bacterial adhesion, a critical factor in preventing secondary caries. In the field of endodontics, two‐photon polymerization has been used to print polymeric microfluidic networks within root canal fillers. These networks enable the continuous delivery of calcium hydroxide, maintaining a pH > 10.5 for 90 days to prevent reinfection.^[^ ^246^, ^247^ ^]^

The advent of 4D printing has introduced a new dimension to dental restorative materials. The 4D printed scaffold can realize accurate shape recovery triggered by an external magnetic field through the magnetic deformation and thermal/magnetic dual‐driving mechanism of magnetic response material in the magnetic field (Figure 7ii(a)). This mechanism is based on the conversion of magnetic field energy to induce reversible phase transition or molecular chain rearrangement within the material (Figure 7ii(b)).^[^ ^67^ ^]^ However, the slow print speeds and high costs associated with two‐photon polymerization and 4D printing currently limit their use to research and niche applications.^[^ ^248^ ^]^

Powder bed fusion techniques, such as selective laser sintering (SLS) and selective laser melting (SLM), excel in processing metals and high‐performance ceramics. CoCr alloys fabricated via SLM exhibit exceptional fatigue resistance, making them ideal candidates for removable partial denture frameworks.^[^ ^249^ ^]^ For ceramics, laser‐sintered 3Y‐TZP zirconia achieves near‐theoretical density with minimal low‐temperature degradation, as shown in SEM images showing microdefects (Figure 7iii), which is a critical advantage for ensuring long‐term clinical performance.^[^ ^89^, ^97^, ^100^, ^191^ ^]^ Laser‐induced forward transfer, a novel extension of powder bed fusion, enables multi‐material printing by sequentially depositing ceramic and polymer inks.^[^ ^242^ ^]^ This technique has been successfully used to create a graded DEJ with modulus transitions that effectively reduce stress concentrations at the interface, a key factor in preventing restoration failure.

Material extrusion methods, such as direct ink writing (DIW), offer unique advantages for the fabrication of bioactive and porous structures. DIW‐printed calcium phosphate cements blended with chitosan not only achieve suitable compressive strengths but also promote osteogenesis, as evidenced by elevated ALP activity levels.^[^ ^83^ ^]^ These scaffolds, with controlled pore sizes in the range of 200–600 µm, facilitate vascular ingrowth in vivo, which is a critical factor in bone regeneration.^[^ ^250^, ^251^ ^]^ However, the slow extrusion rates and challenges in maintaining ink rheology during printing hinder their scalability for high‐volume clinical use. Binder jetting provides a rapid production method for porous temporary restorations by depositing liquid binders onto ceramic or polymer powder beds.^[^ ^81^ ^]^ Post‐processing steps, such as sintering or infiltration, can enhance mechanical properties, but they may introduce dimensional inaccuracies, limiting their use to non‐critical applications.

To overcome the individual limitations of AM and conventional techniques, a hybrid approach can be adopted. For example, combining DLP‐printed zirconia cores with pressed lithium disilicate veneers leverages the strength of the former and the aesthetics of the latter, resulting in restorations that rival natural teeth in both form and function.^[^ ^252^ ^]^ Surface functionalization techniques, such as atomic layer deposition (ALD), can further augment AM outputs by reducing water sorption and extending the service life of the restorations in the saliva‐rich oral environment.^[^ ^253^ ^]^

Despite the significant advancements in AM, several challenges persist. Residual stresses generated during layer‐wise fabrication, high equipment costs, and regulatory ambiguities are some of the key issues.^[^ ^254^ ^]^ However, emerging solutions are on the horizon. The incorporation of closed‐loop feedback systems and self‐healing materials is driving AM toward smarter, greener manufacturing, which will ultimately lead to more efficient and sustainable production of dental restorative materials.^[^ ^255^ ^]^

### 5.2 Refined and Reliable Subtractive Manufacturing

Subtractive manufacturing, especially computer‐aided design/computer‐aided manufacturing (CAD/CAM), has firmly established itself as a cornerstone of dental restoration. This is due to its remarkable capability to produce high‐strength, precision‐engineered prosthetics from monolithic materials.^[^ ^9^, ^142^, ^249^, ^256^ ^]^

**Figure 8:**
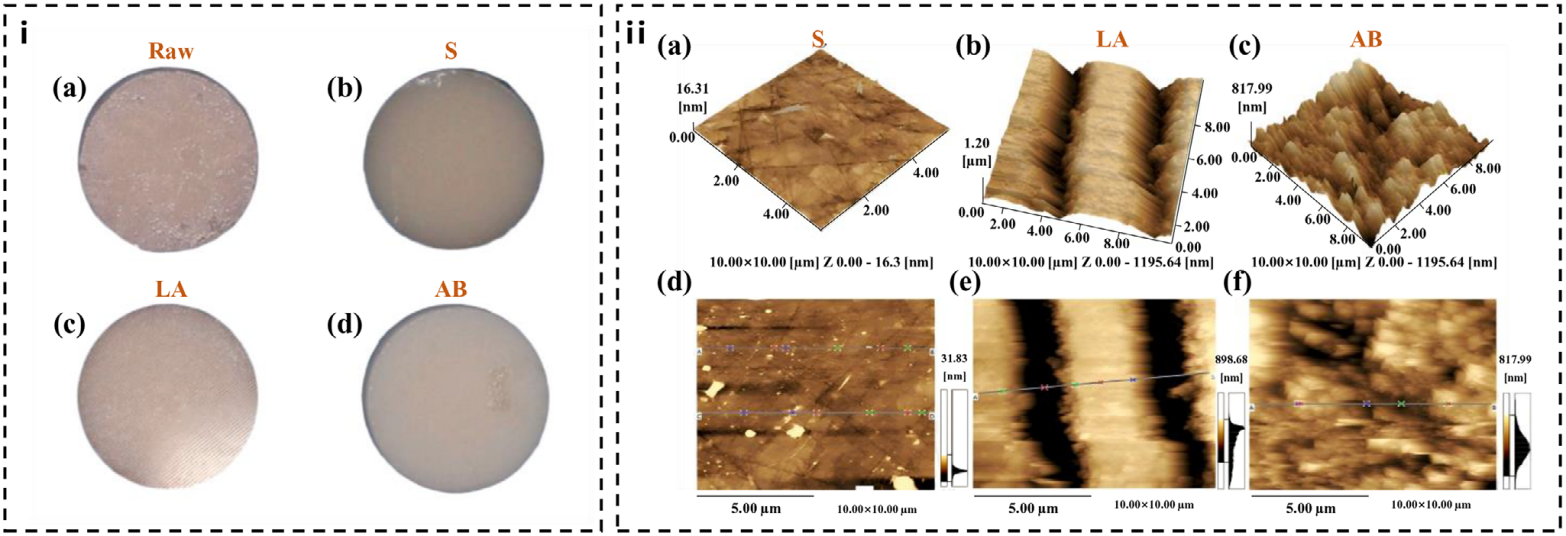
Zirconia surface microstructure and roughness analysis. (i) The macrograph of zirconium tablets with each surface roughing treatments. a) The front view of Y‐TZP before polishing. b) The Y‐TZP experienced polishing. c) The front femtosecond UV laser etched Y‐TZP. d) The surface image of Y‐TZP after air blasted + acid etched. (ii) Surface photography and roughness of femtosecond laser ablation produced ultra‐fine grooves on zirconia materials. (a) (b) (c) Plane profiles of S, LA and AB group in order by AFM. (d) e,f) 3D profiles of S, LA and AB group in order by atomic force microscopy (AFM). Reproduced with permission.^[^ ^260^ ^]^ Copyright 2022, Elsevier.

Modern 5‐axis milling systems are capable of achieving exceptional dimensional accuracy and surface finish, which are indispensable for optimal biomechanical performance and longevity.^[^ ^257^, ^258^ ^]^ The technology demonstrates outstanding performance in processing high‐performance ceramics such as lithium disilicate and yttria‐stabilized zirconia. Advanced milling strategies that incorporate real‐time force feedback and adaptive toolpaths have substantially improved processing efficiency and tool longevity.^[^ ^252^, ^259^ ^]^ However, material waste remains a significant drawback, which has spurred innovations in nesting algorithms and block geometry optimization. Recent developments have successfully integrated milling with secondary processes to enhance functionality. Femtosecond laser ablation enables surface polishing to sub‐micron roughness while preserving mechanical properties, resulting in nearly eliminating Streptococcus mutans biofilm formation while preserving mechanical strength.^[^ ^260^ ^]^ **Figure** **8**i systematically demonstrates the surface evolution of Y‐TZP discs under different processing methods: raw discs exhibit uneven matte surfaces (Figure 8i(a)), while polished discs achieve mirror‐like smoothness (Figure 8i(b)). Notably, femtosecond laser ablation creates periodic microgrooves with untreated smooth ridges (Figure 8i(c)), contrasting with the irregular roughness from air blasting+acid etching (Figure 8i(d)). Atomic force microscopy (AFM) in Figure 8ii reveals distinct topographical features: the laser‐treated group (LA) shows regular groove/ridge structures with ridge width ≈2.39 µm (Figure 8ii(b,e)), while the air‐abraded group (AB) presents random protuberances (Figure 8ii(c,f)).

Hybrid systems combining milling with laser texturing create microretentive surface patterns that significantly promote osseointegration.^[^ ^261^, ^262^, ^263^ ^]^ The evolution of chairside CAD/CAM systems has revolutionized same‐day restorations, with AI‐powered occlusion analysis substantially reducing clinical adjustment time. However, material limitations persist, particularly regarding wear resistance and bond strength to tooth structures. Resin‐ceramic hybrids exhibit wear rates of 100–150 µm per year, exceeding natural enamel (20–30 µm per year), and their bond strength to dentin (15–20 MPa) lags behind traditional resin composites.^[^ ^12^, ^94^ ^]^ As subtractive and additive manufacturing converge in hybrid platforms, CAD/CAM milling will continue to maintain its dominance for high‐strength monolithic restorations while adapting to support multi‐material, biofunctional designs.

### 5.3 Advanced Electrospinning and Nanofiber Architectures for Biomimetic Dental Regeneration

**Figure 9:**
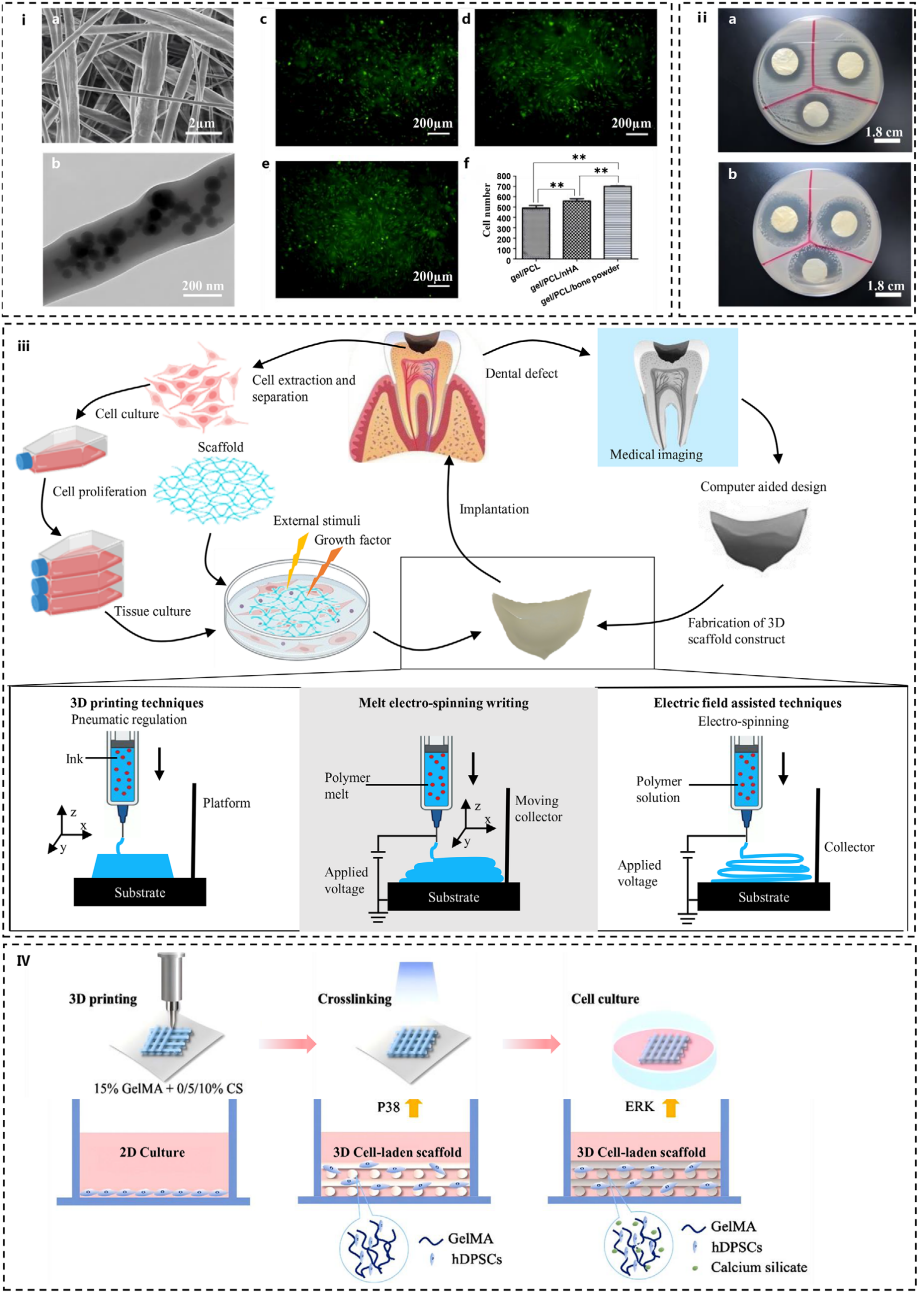
Electrospinning and bioprinting technology. (i) Morphological characterization and cell viability analysis of ADSCs seeded on the three types of scaffolds after 48 h. a) SEM images of gelatin/PCL/nHA. b) TEM images of gelatin/PCL/nHA. c) Cells on gelatin/PCL fiber mat. d) Cells on gelatin/PCL/nHA fiber mat; e) Cells on gelatin/PCL/bone powder fiber mat. f) Counting of living cells on scaffold and comparison of each group. Reproduced under the terms of the CC BY 4.0 license. Permission obtained from the MDPI, Basel, Switzerland.^[^ ^265^ ^]^ Copyright 2016, Dongming Rong et al. (ii) Antibacterial activity of tetracycline hydrochloride‐loaded 10% (w/w) nHA/PCL membranes (triplicate samples). Diameter of inhibition zone against (a) E. coli (2.53 ± 0.06 cm) and (b) B. cereus (2.87 ± 0.06 cm); diameter of nanofibers  =  1.8 cm. Reproduced with permission.^[^ ^271^ ^]^ Copyright 2017, Springer. (iii) The integration of bioprinting and manufacturing technologies. (iv) Schematic diagram depicting the fabrication of CS/GelMa scaffolds and advantages of hDPSCs‐laden CS/GelMa scaffolds for odontogenesis. Reproduced under the terms of the CC BY 4.0 license. Permission obtained from the MDPI, Basel, Switzerland.^[^ ^110^ ^]^ Copyright 2021, Yi‐Ting Lin etal.

Electrospinning has evolved beyond simple fiber fabrication into a multiscale design platform for dental restorative materials, enabling precise recapitulation of native tissue hierarchies.^[^ ^264^ ^]^ By decoupling the contributions of fiber topology (diameter, alignment) and biochemical cues (bioactive doping), this technique offers unique opportunities to engineer cell‐microenvironment interactions, as demonstrated in **Figure** **9**i where SEM/TEM imaging and Live/Dead staining at 2 days after cell seeding reveal fabricated compsite fiber mats promoting robust adipose‐derived mesenchymal stem cells (ADSC) adhesion and viability, directly validating topological optimization for stem cell compatibility.^[^ ^265^ ^]^ Recent mechanistic studies reveal that sub‐500 nm fiber diameters preferentially activate integrin‐mediated focal adhesion kinase signaling in DPSCs, while aligned fibers (1–2 µm spacing) induce contact guidance via RhoA/ROCK cytoskeletal remodeling.^[^ ^216^, ^217^, ^257^ ^]^ These findings rationalize the superior osteogenic differentiation (3–5 fold upregulation of RUNX2/OCN) observed on aligned PCL/nHA scaffolds in periodontal defect models.^[^ ^120^ ^]^

Core‐shell nanofiber designs represent a paradigm shift in functional dental materials, combining mechanochemical gradients with spatiotemporal drug release. Coaxial electrospinning of PCL (shell) and nHA (core) achieves a biomimetic modulus matching dentin's viscoelastic response, while sustained Ca^2^⁺ release (0.5–1.2 mM per day) triggers DPSC mineralization via calcium‐sensing receptor activation.^[^ ^107^, ^266^, ^267^, ^268^ ^]^ Antimicrobial functionality is engineered through innovative release mechanisms: metronidazole‐loaded chitosan fibers exploit bacterial protease‐triggered degradation for on‐demand antibiotic release, achieving >90% P. gingivalis inhibition at sub‐MIC concentrations (Figure 9ii).^[^ ^123^, ^128^, ^269^ ^]^

The convergence of electrospinning with additive manufacturing is redefining scaffold design paradigms, as visualized in Figure 9iii where hybrid 3D‐printed frameworks with electrospun interlayers replicate the dentin enamel junction's modulus gradient (40–60 MPa compressive strength), with finite element modeling confirming stress distribution akin to natural teeth, bridging macroscale printing precision with nanoscale biochemical functionality.^[^ ^265^, ^270^, ^271^ ^]^ Emerging 4D electrospinning systems incorporate stimuli‐responsive polymers (e.g., pH‐sensitive chitosan) that autonomously adapt fiber swelling ratio to release antimicrobials in cariogenic microenvironments.^[^ ^20^, ^61^, ^67^, ^272^ ^]^

Despite progress, fundamental challenges persist in translational scalability. Non‐Newtonian jet dynamics during electrospinning cause fiber heterogeneity under±5 kV fluctuations, requiring machine learning‐based real‐time voltage control to maintain <5% diameter variations.^[^ ^273^ ^]^ Industrial adoption demands innovations like high‐throughput multi‐needle arrays with electrostatic field focusing, currently achieving 20 g/h production rates at 80% fiber uniformity.^[^ ^148^, ^274^ ^]^ Future directions include CRISPR‐edited cell‐selective nanofibers and AI‐optimized topology for patient‐specific periodontal regeneration.^[^ ^71^, ^111^, ^138^, ^140^, ^275^, ^276^ ^]^

### 5.4 Multiscale Hybrid Manufacturing: Bridging Material Interfaces and Biological Functionality

The convergence of disparate manufacturing modalities is redefining dental restorative design through atomic‐to‐macroscale control, where interfacial engineering and biological orchestration emerge as critical determinants of clinical success. Microwave‐assisted hybrid sintering exemplifies this paradigm, where dielectric polarization at GHz frequencies (2.45–5.8 GHz) selectively excites ionic defects in 45S5 Bioglass, inducing homogeneous nucleation of apatite‐wollastonite phases with 50–100 nm crystallite sizes—a key factor in achieving fracture toughness (3.0 MPa·m^1^^/^^2^) rivaling natural dentin.^[^ ^97^, ^277^ ^]^ In situ synchrotron XRD studies reveal that microwave's non‐thermal effects lower crystallization activation energy by 15–20%, enabling rapid densification while suppressing deleterious phase segregation.^[^ ^278^ ^]^ When integrated with robocasting, this approach creates functionally graded restorations with spatially tuned crystallinity (20–80 vol %), mimicking the enamel‐dentin junction's property transition through computational topology optimization.^[^ ^279^ ^]^ The direct energy coupling mechanism promotes uniform crystallization while minimizing thermal gradients that compromise interfacial integrity.

Multi‐material integration systems now transcend simple mechanical bonding, leveraging atomic‐scale interfacial engineering to direct biological responses. ALD of 10–20 nm Al_2_O_3_ interlayers on DLP‐printed zirconia frameworks increases interfacial fracture energy by 300% through oxygen vacancy passivation, while plasma electrolytic oxidation generates microporous TiO_2_ surfaces that upregulate integrin α_2_β_1_ expression in gingival fibroblasts—enhancing soft‐tissue sealing.^[^ ^253^, ^280^ ^]^ These advances address the longstanding “material mismatch” challenge in hybrid prostheses, where conventional veneering techniques suffer from 20–30% delamination rates under mastication loads.^[^ ^252^, ^281^ ^]^

Bioprinting has evolved from cell‐laden hydrogel deposition to spatially programmable tissue morphogenesis. Figure 9IV schematically illustrates the fabrication of CS/GelMa scaffolds, where hDPSCs‐laden constructs demonstrate triphasic regeneration, direct evidence of bioprinting's evolution from hydrogel deposition to programmable tissue morphogenesis.^[^ ^110^ ^]^ Recent breakthroughs utilize microfluidic printheads to co‐print DPSCs (10⁶ cells/mL) with nHA‐doped GelMA (15% w/v) and vascular endothelial growth factor (VEGF)‐loaded PLGA microparticles (5–10 µm diameter), creating triphasic constructs that simultaneously regenerate dentin‐like tissue (DMP‐1), vascular networks (CD31), and neural sprouts (β‐III tubulin) in rodent models.^[^ ^110^, ^275^, ^282^ ^]^ Light‐based bioprinting achieves single‐cell precision through two‐photon polymerization (780 nm femtosecond laser) of glycidyl methacrylate‐hyaluronic acid bioinks, patterning 20–50 µm capillary precursors that anastomose with host vasculature within 7 days.^[^ ^283^ ^]^ Machine learning now optimizes printing parameters in silico, reducing empirical testing by 90% while predicting cell viability for complex geometries.^[^ ^284^ ^]^

The frontier lies in intelligent biohybrid systems. 4D‐printed shape‐memory polyurethane scaffolds with NiTi reinforcement (30 vol %) autonomously adapt to root canal anatomy upon body temperature activation, while eluting stromal cell‐derived factor‐1 to recruit endogenous stem cells.^[^ ^138^, ^285^, ^286^ ^]^ For immunomodulation, IL‐4‐functionalized nanocellulose bioinks (7.5% w/v) induce M2 macrophage polarization via signal transducer and activator of transcription 6 signaling, reducing fibrotic capsule thickness by 60% in porcine gingival models.^[^ ^67^, ^111^, ^287^, ^288^ ^]^ However, regulatory hurdles persist for living constructs—current FDA guidelines lack standards for viability thresholds or functional metrics.

Future directions demand closed‐loop manufacturing. Embedded biosensors (pH, O_2_, strain) in 3D‐printed scaffolds enable real‐time monitoring via wearable radio frequency identification readers, while AI‐driven adaptive printing adjusts bioink composition based on sensor feedback—an approach recently demonstrated for diabetic wound dressings but yet unexplored in dentistry.^[^ ^173^ ^]^

### 5.5 Intelligent Dental Restorations: From Atomic Programming to Environmental Adaptation

The next revolution in dental materials lies in systems that transcend static functionality, instead exhibiting life‐like responsiveness through molecular programming and environmental cognition 4D printing exemplifies this shift, where temporal control is achieved via precisely engineered phase transitions—polyurethane networks with oligo (ε‐caprolactone) switches autonomously adapt to root canal anatomy within 2–3 min of intraoral placement, generating 0.15–0.3 MPa interfacial stress to seal microgaps without clinician intervention.^[^ ^272^ ^]^ Mechanistically, this shape‐memory behavior arises from crystallite melting entropy and strain‐induced nucleation of auxetic microstructures, as revealed by in situ synchrotron radiation small angle/wide‐angle X‐ray scattering.^[^ ^20^ ^]^

Stimuli‐responsive hydrogels achieve spatiotemporal therapeutic precision through multi‐stage triggering. For example, chitosan‐grafted poly hydrogels are dual‐responsive: at pH < 5.5 they swell 400% to release ACP, while at 37 °C they collapse hydrophobically to reinforce the lesion site.^[^ ^64^, ^106^, ^125^ ^]^ Molecular dynamics simulations show this pH‐dependent swelling correlates with protonation‐induced charge neutralization, enabling 70 µm enamel remineralization in 14 days—3× faster than passive diffusion systems.^[^ ^36^, ^124^ ^]^

**Figure 10:**
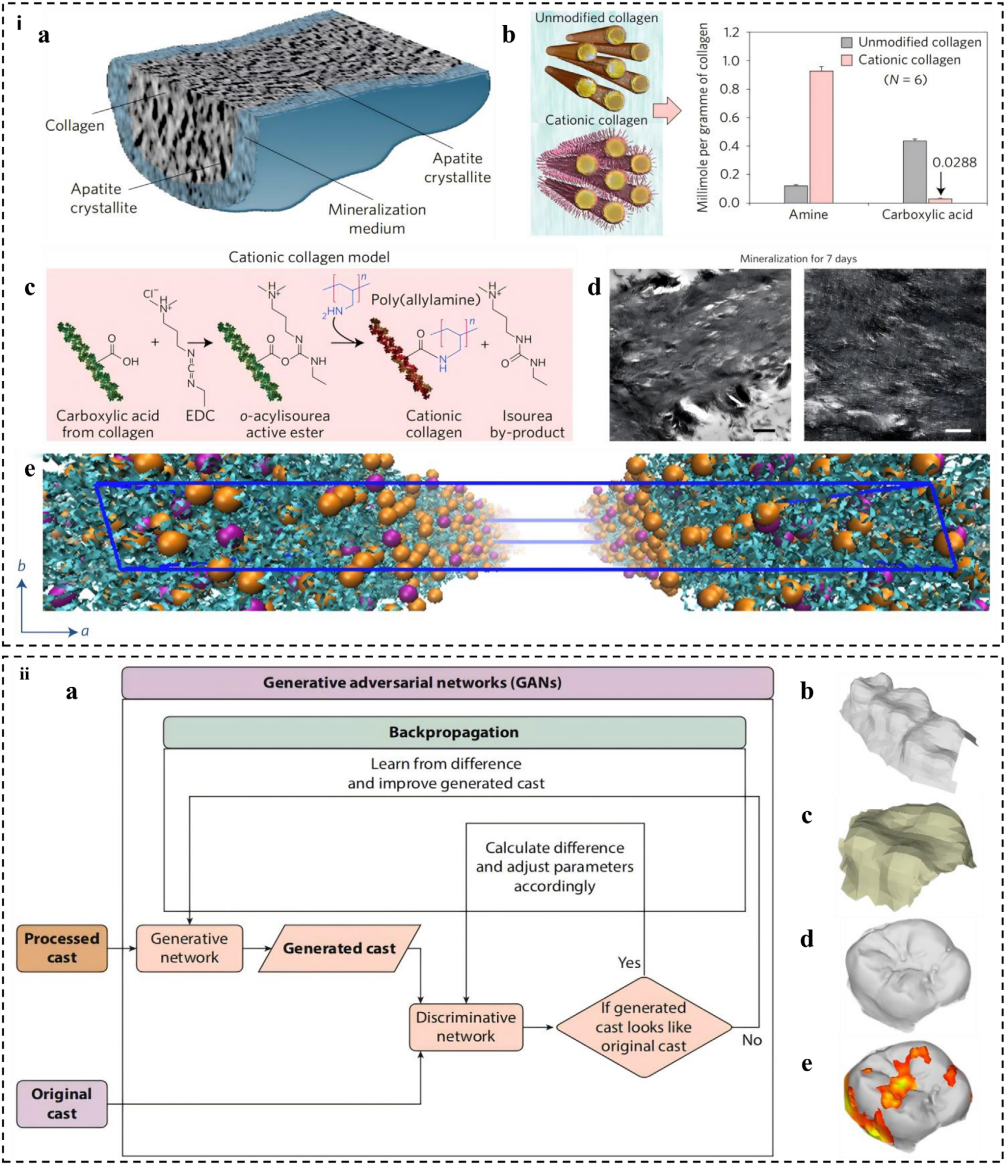
Emerging techniques of dental restorative biomaterials. (i) Cationic collagen model of PAH‐ACP intrafibrillar mineralization. a) Visualization of 3D volume of one part of the mineralized fibril. b) Method of conjugation of polycations to collagen fibrils via amino acids containing –COOH side chains. EDC: carbodiimide. c) Comparison of free amine and carboxyl groups between cationic and unmodified collagen. The error bars show standard deviations of the measurements. Decrease in carboxyl groups is due to their conjugation with PAH via the O = C−N−H linkage. d) TEM of leaflets from cationic collagen sponges mineralized by PAH‐ACP for 7 days (Left bar, 500 nm. Right bar, 200 nm). Heavily mineralized cross‐banded collagen could be identified. e) Representation of the collagen structures with intrafibrillar mineralization precursors (simplified as Ca ions) in the polycationic system. Blue ribbons represent collagen structures and orange and purple dots presents Ca ions present in the intrafibrillar regions. Orange‐coloured dots represent Ca ions in the 0.21–0.425 nm region, and purple‐colored dots represent Ca ions in the 0.425–0.625 nm region from the collagen structures. Reproduced with permission.^[^ ^293^ ^]^ Copyright 2017, Springer Nature. (ii) Accuracy of artificial intelligence‐designed single‐molar dental prostheses. (a) Architecture of Generative Adversarial Network (GAN). (b) Part of generated cast. (c) AI‐designed tooth isolated from generated cast. (d) Original tooth. Demonstration of comparing AI‐designed tooth to it. Hausdorff distance presented by color (relative measurement for each pair). (e) Demonstration of comparing AI‐designed tooth to it. Lowest Hausdorff distance in red (0.000 mm). Highest Hausdorff distance in blue (1.500 mm). Reproduced with permission.^[^ ^294^ ^]^ Copyright 2022, Elsevier.

Nanoparticle self‐assembly has progressed from structural mimicry to atomic‐scale biomimicry. DNA origami‐directed hydroxyapatite (HAp) mineralization achieves 2 nm positional accuracy by programming Ca^2+^ nucleation sites via phosphate‐modified oligonucleotides, yielding enamel‐mimetic prisms with Vickers hardness (3.5–4.0 GPa) matching natural enamel.^[^ ^99^, ^289^, ^290^, ^291^, ^292^ ^]^ Mineralization within collagen polymer fibers can be guided by the use of polycations and polyanions. Figure 10i(d) visualizes this process through TEM of mineralized collagen sponges after 7 days, revealing heavily cross‐banded collagen with intrafibrillar mineralization—a direct validation of the cationic collagen model (**Figure** **10**i(a–e)).^[^ ^293^ ^]^ Cryo‐ET reveals these assemblies replicate the interrod protein matrix's role, with amelogenin‐mimetic peptides guiding HAp nanocrystal alignment within 5° angular deviation—surpassing natural biomineralization precision.^[^ ^289^ ^]^

AI‐driven manufacturing is transforming every aspect of dental fabrication, with Figure 10ii exemplifying its precision in single‐molar prostheses. Generative adversarial networks (GANs; Figure 10ii(a)) design graded material architectures that optimize stress distribution, while Hausdorff distance mapping Figure 10ii(e) demonstrates sub‐millimeter accuracy (0.000–1.500 mm) compared to original teeth—reducing post‐sintering failure rates from 15% to 2% when combined with convolutional neural networks for defect detection.^[^ ^294^ ^]^ Convolutional neural networks trained on SEM images detect subsurface defects in zirconia crowns with high accuracy, reducing post‐sintering failure rates from 15% to 2%.^[^ ^295^ ^]^ Generative adversarial networks design graded material architectures, optimizing stress distribution and enhancing the overall performance of the restorations.^[^ ^296^ ^]^ Reinforcement learning algorithms autonomously adjust laser parameters during selective laser melting, achieving high‐density alloys with reduced energy consumption.^[^ ^297^, ^298^ ^]^ Environmentally conscious manufacturing has become increasingly sophisticated, with closed‐loop material recycling systems and bio‐based resin formulations reducing the ecological footprint of dental restoration.^[^ ^299^ ^]^ Closed‐loop recycling systems reclaim unused zirconia powder, coupled with AI‐powered nesting algorithms that minimize material waste during CAD/CAM milling.^[^ ^300^ ^]^ These approaches demonstrate that clinical excellence and environmental responsibility need not be mutually exclusive.

AI has rapidly advanced from concept to practice in dental material research (**Table** **4**). Recent studies have demonstrated its ability to automate prosthetic design,^[^ ^301^, ^302^ ^]^ accurately predict composite performance across mechanical, optical, and tribological properties,^[^ ^303^, ^304^, ^305^, ^306^, ^307^, ^308^ ^]^ and enable high‐throughput optimization through interpretable and inverse design approaches.^[^ ^303^, ^307^ ^]^ In parallel, AI‐based analytical tools have improved detection of degradation products and forecasting of long‐term stability.^[^ ^307^, ^309^ ^]^ Collectively, these advances highlight AI as a powerful driver of multiscale dental material discovery, bridging composition, performance, durability, and clinical translation.

**Table 4:** Representative studies demonstrating AI applications in dental material discovery and multiscale design.

Mapped section	Method/Model	Application	Detailed results	Refs.
Prosthetic design and clinical imaging	3D‐CNN^a)^ + CAD	Partial crown synthesis	17‐layer 3D‐CNN generated crowns from intraoral scans; ≈60% validation accuracy; proof‐of‐concept for workflow automation	[301]
	SqueezeNet + Logistic Regression	Veneer finish line detection	>90% accuracy; logistic regression better than naïve Bayes; clinical imaging outperformed traditional inspection	[302]
Composition and property prediction; Optimization	Interpretable ML^b)^ (RF^c)^, GBDT^d)^, LightGBM)	Composite property prediction	R^2^ = 0.93–0.99; filler, TEGDMA, UDMA major contributors; predicted new high‐strength compositions (>260 MPa)	[303]
Composition and property prediction	Multi‐model ML (SVM^e)^, RF, KNN^f)^, XGBoost, etc.)	Large dataset composite predictions	Best classifiers varied by property (e.g., KNN for modulus, RF/XGBoost for shrinkage); identified BisGMA, UDMA, filler, depth of cure as key drivers	[305]
Composition and property prediction; Degradation	XGBoost^g)^, AdaBoost^h)^, RF, KNN	Mechanical degradation prediction	AdaBoost best for flexural strength (R^2^ = 0.99); XGBoost best for hardness (R^2^ = 0.99); immersion time dominant factor	[304]
Property prediction; Degradation	ANN^i)^ (Bayesian Reg., LM^l)^, SCG^j)^)	Abrasive wear prediction	R^2^ up to 0.997; immersion time and load strongest predictors; SEM confirmed predicted wear patterns	[306]
Property prediction; Degradation	MLP^k)^, XGBoost, KNN	Wear progression prediction	XGBoost achieved R^2^ = 0.9996; average error <1%; immersion time & composition most important	[310]
Degradation and stability	Logistic Regression	Degradation analysis (LC‐MS/MS)	Identified 21 monomers/degradation products (e.g., BisGMA, UDMA, TEGDMA); provided probability‐based confidence	[309]
Composition and property prediction; Optimization; Degradation	ExtraTrees	Creep performance prediction	R^2^ = 0.9999; ORMOCER^o)^ and SiO_2_ strongest contributors to creep recovery; 647700 formulations screened	[307]
Optimization and inverse design	CNN + Genetic Algorithm	Composite microstructure optimization	CNN predicted modulus, strength, toughness with <5% error; GA discovered optimal stiff/tough microstructures	[308]

^a)^ Convolutional neural network;

^b)^ Machine learning;

^c)^ Random forest;

^d)^ Gradient boosting decision tree;

^e)^ Support vector machine;

^f)^ K‐Nearest neighbors;

^g)^ eXtreme gradient boosting;

^h)^ Adaptive boosting;

^i)^ Artificial neural network;

^j)^ Scaled conjugate gradient;

^k)^ Multi‐layer perceptron);

^l)^ Organically modified ceramic.

The frontier lies in biohybrid intelligent systems. 3D‐printed graphene oxide/polyaniline biosensors embedded in restorations detect MMP‐8 proteases (caries biomarker) with 0.1 pM sensitivity, wirelessly alerting clinicians via intraoral IoT networks.^[^ ^145^ ^]^ Critical challenges remain in standardizing dynamic material testing and establishing FDA pathways for AI‐designed devices. Future systems may integrate quantum dot‐based pH mapping and CRISPR‐edited biofilm sensors, heralding an era of truly “smart” dental ecosystems.

## 6 Multiscale Optimization Paradigms: Bridging Bioinspired Design to Clinical Realities

**Figure 11:**
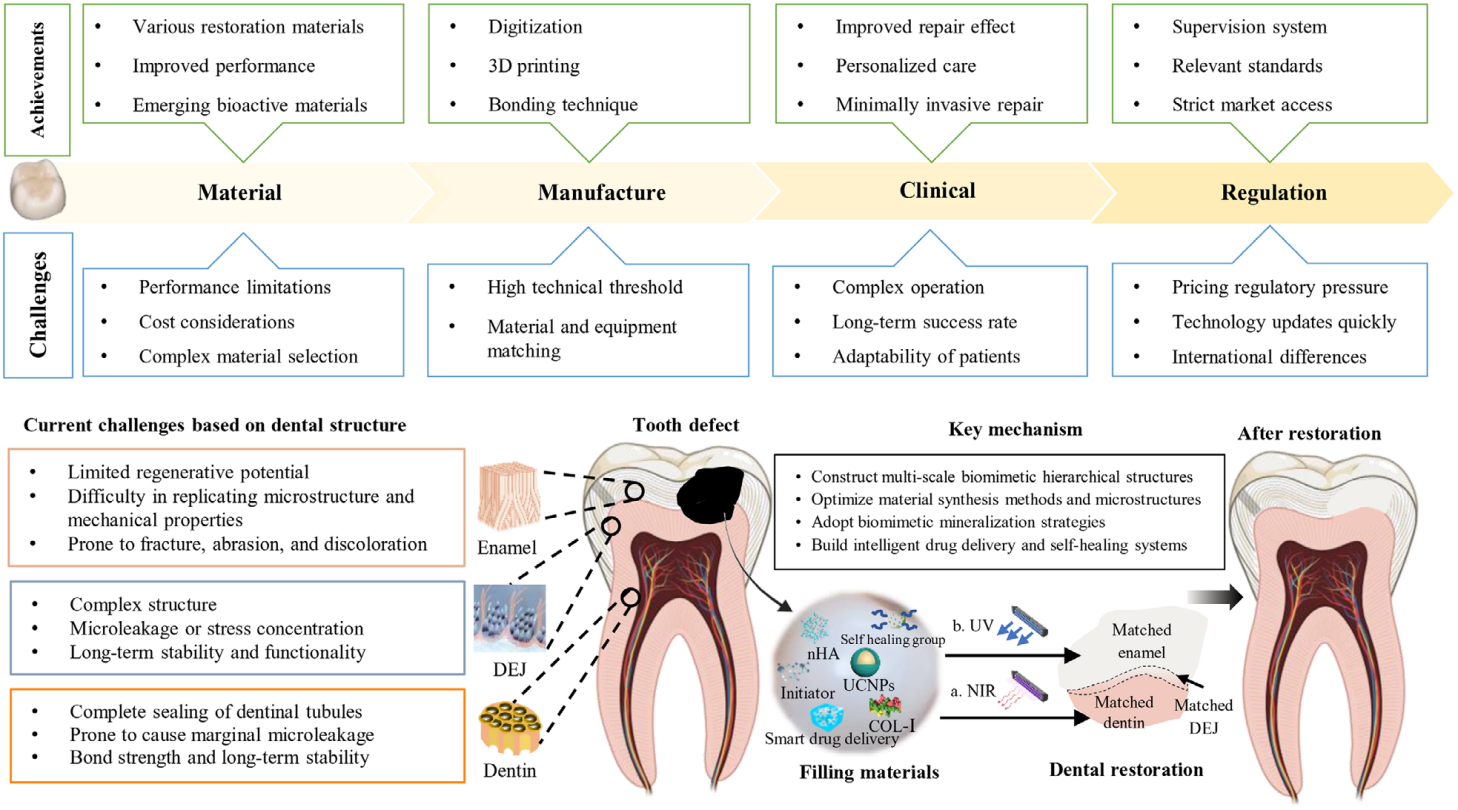
Challenges exist in dental restorative materials. DEJ: dentinoenamel junction; UV: ultraviolet; NIR: near‐infrared rays; UCNPs: upconversion nanoparticles; nHA: nano‐hydroxyapatite; COL‐I: type I collagen. The designed functional material for restoring dental defects undergoes an initial step: it is irradiated with NIR to attain a structure and performance that match those of internal dentin, followed by a transformation to align with the DEJ. Subsequently, bit is irradiated with UV light for external curing, achieving a structure and performance akin to enamel.

Optimized dental restoratives, including zirconia‐resin hybrids and functionally graded PICN, are summarized in **Table** **5**. These materials represent advancements in atomic‐to‐macroscale biointerfacial programming, dynamic biological responsiveness, and AI‐driven performance prediction. As illustrated in **Figure** **11**, in response to current challenges in material selection, processing, clinical translation, and regulatory compliance for dental structures and restorative materials, the following multi‐scale optimization strategies are proposed. Based on these strategies, a transformation sequence has been mapped to simultaneously achieve mechanical durability, integration of biological activity, and antibacterial functionality compatible with the oral microbial community.

**Table 5:** Optimized dental restorative materials.

Category	Design strategy	Mechanical property	Bioactivity	Antimicrobial efficacy	Clinical applicability	Refs.
Zirconia‐resin hybrid	10‐MDP^a)^ silanization + nano‐ZrO_2_ filler	Flexural strength: 350–450 MPa	HAP^b)^ formation at interface	N/A^c)^	Posterior crowns, bridges	[12, 94, 152, 311]
Functionally graded PICN^d)^	UDMA^e)^‐infiltrated porous ceramic gradient	Modulus: 20 GPa (surface) → 2 GPa (core)	Strain‐adaptive stress dissipation	N/A	Full‐arch prostheses	[94, 96]
GO^f)^‐reinforced composite	Aligned graphene oxide nanosheets (0.3 wt %)	Fracture toughness: 2.5 MPa·m^1^/^2^	N/A	N/A	High‐stress restorations	[83, 177, 312]
pH‐responsive hydrogel	Poly(acrylic acid‐co‐NVP^g)^) + ACP^h)^ nanoparticles	Compressive strength: 50–80 MPa	Ca^2^⁺/PO_4_^3−^ release (pH <5.5)	N/A	Caries‐inhibiting liners	[103, 176, 177]
QAS‐NP^i)^ modified resin	Covalent QAS‐NP integration	Flexural strength: 90–120 MPa	N/A	70% S. mutans reduction	Class I/II dental restorations	[9, 127]
DLC^j)^‐coated alloy	3–5 µm diamond‐like carbon coating	Hardness: 15–20 GPa	N/A	90% wear reduction	Implant abutments, occlusal surfaces	[102]

^a)^ 10‐methacryloyloxydecyl dihydrogen phosphate;

^b)^ Hydroxyapatite;

^c)^ Not applicable;

^d)^ Polymer‐infiltrated ceramic networks;

^e)^ Urethane dimethacrylate;

^f)^ Graphene oxide;

^g)^ N‐vinyl pyrrolidone;

^h)^ Amorphous calcium phosphate;

^i)^ Quaternary ammonium salt nanoparticles;

^j)^ Diamond‐Like Carbon.

### 6.1 Atomic‐to‐Macroscale Biointerfacial Engineering

Interface engineering has evolved from passive bonding to active biomolecular recognition. Phosphate‐based silane coupling agents now leverage Zr‐O‐P covalent bonds (bond energy ≈532 kJ mol^−1^) with zirconia surfaces, achieving 45 MPa shear strength retention after 50 000 thermocycles—surpassing conventional MDP primers by 300% in hydrolysis resistance.^[^ ^311^, ^313^ ^]^ For dentin hybridization, MMP‐inhibiting adhesives like methacryloyloxyethyl‐phosphorylated choline employ dual‐action mechanisms: quaternary ammonium groups electrostatically block MMP‐2/9 catalytic domains, while phosphate‐calcium chelation stabilizes collagen fibrils at the nanofibril‐mineral interface.^[^ ^145^, ^314^, ^315^ ^]^

Bioactive glass interfaces have entered the “iontronic” era, where pulsed Ca^2^⁺/PO_4_^3−^ release profiles are precisely tuned to match odontoblast activity rhythms via borate glass network modifiers.^[^ ^124^ ^]^ Synchrotron micro‐CT reveals these designs form 50–200 nm hydroxyapatite bridges at restoration margins within 7 days, reducing the risk of secondary caries though the synergistic effect of pH buffering, destruction of biofilm EPS, and selective remineralization of carious dentin.^[^ ^30^, ^31^, ^239^ ^]^

Building on DEJ mechanoadaptation principles, FGMs achieve enamel‐like modulus (20 GPa) at the surface and dentin‐like compliance (2 GPa) internally. This gradient minimizes stress shielding at the DEJ under cyclic masticatory forces, outperforming homogeneous zirconia restorations in 5‐year clinical survival rates.^[^ ^11^, ^316^ ^]^ PICNs exemplify this approach, combining ceramic strength with polymer toughness to mimic natural tooth biomechanics, effectively dissipating occlusal forces while maintaining aesthetic translucency.^[^ ^96^ ^]^ Clinical studies report 92% survival rates for PICN‐based crowns at one year, comparable to lithium disilicate but with superior fracture resistance in high‐stress scenarios.^[^ ^317^ ^]^ These designs significantly enhance fatigue resistance, a critical requirement for long‐term clinical success. The biological rationale for gradient designs extends beyond mechanical performance. The gradual transition in elastic modulus minimizes stress shielding, a phenomenon where stiff materials redirect stresses to weaker adjacent tissues, potentially leading to bone resorption or enamel microfractures. By closely approximating the natural tooth's mechanical behavior, gradient designs promote physiological load distribution, thereby preserving the integrity of surrounding tissues.

Nanoscale reinforcement strategies address the trade‐off between strength and toughness in dental composites. Aligned graphene oxide nanosheets embedded in resin matrices increase tensile strength and elastic modulus while enhancing wear resistance.^[^ ^312^ ^]^ Core‐shell nanoparticles, such as silica‐coated zirconia, optimize optical and mechanical performance simultaneously, yielding flexural strengths exceeding 120 MPa.^[^ ^266^ ^]^ However, nanoparticle agglomeration at high concentrations can reduce translucency, necessitating surface functionalization to improve dispersion. These nanoscale modifications not only enhance mechanical properties but also influence cellular behavior. For instance, graphene oxide's surface chemistry can modulate fibroblast adhesion and proliferation, with carboxyl‐functionalized surfaces promoting higher cell viability compared to pristine graphene oxide.^[^ ^268^, ^318^ ^]^

### 6.2 Dynamic Adaptability and Antimicrobial Functionality

Dynamic adaptability mechanisms enable restorations to respond to environmental changes, enhancing longevity and functionality. pH‐responsive materials, such as poly(acrylamide‐co‐acrylic acid), swell in acidic environments (pH <5.5), releasing calcium and phosphate ions to remineralize early caries lesions.^[^ ^103^, ^319^ ^]^ These materials maintain compressive strengths of 50–80 MPa while reducing secondary caries incidence in vitro. Self‐healing composites represent another leap forward, incorporating microcapsules (50–100 µm) filled with TEGDMA monomer and dibenzoyl peroxide initiator.^[^ ^64^, ^65^, ^86^, ^145^ ^]^ When cracks propagate under occlusal stress, the capsules rupture, releasing healing agents that polymerize upon contact, achieving crack closure and restoring the material's original strength.^[^ ^65^ ^]^ Recent advances in thermally adaptive polymers, such as SMPUs, allow restorations to soften at body temperature (35–40 °C), conforming to cavity undercuts before hardening to exert 2–5 MPa recovery stresses.^[^ ^119^, ^230^, ^320^ ^]^ This innovation reduces microleakage compared to conventional composites.

Antimicrobial functionalization is critical to combating biofilm formation and secondary caries. Quaternary ammonium nanoparticles covalently bonded to resin matrices disrupt bacterial membranes via electrostatic interactions, reducing Streptococcus mutans adhesion by 70%.^[^ ^127^ ^]^ Photodynamic functionalization with ICG offers on‐demand antimicrobial action.^[^ ^321^ ^]^ Under near‐infrared light (808 nm), ICG generates singlet oxygen (^1^O_2_), achieving 99.9% bacterial kill rates within 5 min—a promising solution for peri‐implantitis management. Enzyme‐responsive systems, integrating bioactive and smart‐responsive themes, release chlorhexidine in the presence of bacterial proteases. Lysozyme‐responsive chitosan‐streptomycin conjugates eradicate biofilms via protease‐triggered degradation, pH‐dependent ion release, and photodynamic singlet oxygen generation.^[^ ^8^ ^]^ These systems minimize disruption to oral microbiota while effectively controlling pathogenic biofilms.

### 6.3 Fatigue and Wear Resistance

Nanotwinned metals revolutionize fatigue resistance through dislocation engineering. CoCrMo alloys with hierarchical nanotwins (5–20 nm spacing) exhibit 650 MPa fatigue limits, double conventional alloys. This is achieved by three cooperative mechanisms: twin boundary‐induced dislocation blocking, cyclic strain delocalization, and deformation‐induced phase transition.^[^ ^322^ ^]^ DLC coatings now incorporate 5–7 nm MoS_2_ interlayers, achieving wear rates of 0.8×10^−6^ mm^3^ Nm^−1^—matching natural enamel's durability while reducing friction coefficients to 0.08 (vs 0.6 for conventional composites).^[^ ^102^ ^]^

Surface texturing draws inspiration from shark denticle anti‐fouling. Laser‐ablated microgrooves (20 µm width, 5 µm depth) with re‐entrant angles (110–130°) reduce bacterial adhesion by 70% through a combination of minimized contact area, mechanobactericidal effects, and disrupted quorum sensing via topographically hindered cell‐cell contact.^[^ ^129^ ^]^ The biological implications of wear resistance are profound. Excessive wear can lead to tooth structure loss, occlusal disharmony, and temporomandibular joint disorders. By closely mimicking the wear resistance of natural enamel, advanced coatings and surface treatments help maintain occlusal stability and patient comfort over time.

### 6.4 Clinical Translation and Regulatory Challenges

The clinical translation of dental restorative materials demands a balance between biomechanical performance, biological compatibility, and regulatory adherence. Successful restoratives must endure cyclic masticatory forces while maintaining marginal integrity, with gap widths below 50 µm to prevent microleakage.^[^ ^323^, ^324^ ^]^ Biological compatibility requires materials to achieve ISO 10993‐1 cytotoxicity ratings of Grade 1 or lower and limit ion release under acidic conditions.^[^ ^121^ ^]^ Functional integration necessitates mimicking natural teeth's strain‐rate sensitivity and thermal conductivity to avoid pulp irritation, yet many materials falter under the complex interplay of mechanical, chemical, and biological stressors.^[^ ^137^ ^]^ For instance, resin composites with high water sorption initially counteract polymerization shrinkage but degrade over time due to salivary esterases, increasing fracture risk.^[^ ^325^ ^]^

Recent clinical evidence confirms the successful translation of novel restorative materials and techniques into practice (**Table** **6**). Long‐term studies have demonstrated high survival rates for posterior composites, veneers, and zirconia‐based restorations,^[^ ^326^, ^327^, ^328^, ^329^ ^]^ while large practice‐based cohorts have identified key patient‐, tooth‐, and material‐related risk factors.^[^ ^330^ ^]^ Randomized clinical trials further validate the effectiveness of new composites and therapeutic strategies in children and adults,^[^ ^331^, ^332^ ^]^ and systematic reviews confirm that minimally invasive approaches reduce failure risk compared to conventional treatments.^[^ ^333^ ^]^ Collectively, these studies provide quantitative evidence that innovations in dental materials not only perform well in vitro but also demonstrate reliable clinical outcomes across diverse patient populations.

**Table 6:** Quantitative clinical evidence supporting the translation of dental restorative materials.

Material type	Study design	Population	Intervention	Outcome	Clinical relevance	Refs.
Ceramic (feldspathic, lithium disilicate)	Retrospective cohort	341 patients, 1459 veneers	Ceramic veneers	10‐year survival: 89% vs 66%; AFR: 1.2% vs 3.9%	Ceramics show superior long‐term success over composites	[328]
Composite resin	Retrospective cohort (long‐term)	100 patients, 683 posterior restorations	Direct resin composites	33‐year survival 73%; AFR^a)^ <1.1%; failures: fracture, secondary caries	Posterior composites demonstrate decades‐long durability	[329]
	Large practice‐based cohort	14909 patients, 31472 restorations	Composite resin	2‐year survival 85%; higher failure with GI (HR^b)^ 4.07), compomer (HR 4.22)	Identified key patient, tooth, and material risk factors	[336]
Monolithic zirconia	Retrospective study	393 patients, 1143 restorations	Monolithic zirconia	5‐year survival: 100% vs 95.8%; HR 0.34; overall 96.3%	Monolithic zirconia superior longevity vs layered	[327]
Zirconia + lithium disilicate	Prospective clinical trial	25 patients, 25 FDPs	Zirconia FDPs^c)^ veneered with lithium disilicate (CAD‐on)	5‐year survival 100%; all restorations clinically acceptable	CAD‐on FDPs highly reliable, reduced veneer chipping	[326]
Composite resin (bulk‐fill, dual‐cure)	Prospective practice‐based trial	41 patients, 60 restorations	Self‐adhesive bulk‐fill composite	3‐year AFR 3.9%; 83% acceptable; no secondary caries	Promising performance of new bulk‐fill composite	[337]
Silver diamine fluoride (SDF)	RCT^d)^	79 children, 237 teeth	38% SDF^e)^	Caries arrest: 99% vs 94%; treatment time: 3.3 vs 14.4 min	SDF highly effective and efficient in pediatric care	[332]
Alkasite composite	RCT	38 children, 59 molars	Alkasite (Cention N)	1‐year retention 100% both groups; no secondary caries	Both materials clinically effective in pediatric settings	[331]
Composite + ceramic (lithium disilicate, feldspathic, CAD‐CAM composite)	Retrospective case series	8 patients, 212 restorations (severe tooth wear)	Adhesive restorations (veneers)	5‐year survival 90%; ceramic 93% > composite 86%; posterior higher risk	Adhesive rehabilitations effective, with risk stratification	[330]
All‐ceramic (lithium disilicate, zirconia)	Prospective multi‐center cohort	567 patients, 907 implant‐supported crowns	CAD‐CAM all‐ceramic crowns	Success 97% (mean 2.5y); AFR 0.74%; lab > chairside (HR 26)	CAD‐CAM crowns reliable; fabrication technique impacts risk	[333]

^a)^ Annual failure rate;

^b)^ Hazard ratio;

^c)^ Fixed dental prostheses;

^d)^ Randomized controlled trial;

^e)^ Silver diamine fluoride.

Regulatory frameworks further complicate material innovation, with divergent global requirements shaping development pathways. China's NMPA emphasizes chemical stability under extreme pH and temperature cycling to simulate dietary variations. In U.S., FDA mandates comparative performance data against predicate devices for Class II clearance, while EU's MDR enforces stricter post‐market surveillance, requiring 5–10 years of clinical evaluation reports for CE marking, particularly for nanomaterials (particle size <100 nm).^[^ ^150^, ^334^ ^]^ However, standardized testing protocols often inadequately replicate clinical realities, necessitating emerging guidelines that integrate biofilm‐challenge models and thermo‐mechanical cycling to better predict clinical performance.^[^ ^9^, ^335^ ^]^

Innovation faces inherent paradoxes, such as the trade‐off between bioactivity and biostability. Calcium silicate cements promote remineralization but suffer from interfacial instability due to excessive porosity.^[^ ^167^ ^]^ Additive manufacturing, while enabling anatomical precision, grapples with residual monomers that induce chronic inflammation.^[^ ^122^ ^]^ Antimicrobial strategies like nanosilver doping reduce biofilm formation but degrade flexural strength through particle agglomeration.^[^ ^338^, ^339^ ^]^ Addressing these challenges requires a shift toward patient‐specific material ecosystems, leveraging predictive analytics trained on clinical datasets to correlate filler morphology with 15‐year survival rates. Dynamic regulatory frameworks are also evolving, incorporating real‐world evidence from EHRs and material degradation data to iteratively refine safety thresholds.^[^ ^340^ ^]^ Multiphysics models combining electrochemical dissolution and abrasive wear now predict material loss within ±5% of clinical observations, offering a path toward clinically validated designs.^[^ ^341^, ^342^ ^]^ Collaborative initiatives like the FDI World Dental Federation's BioSmart Project aim to harmonize global standards for adaptive materials responsive to pH shifts or mechanical overload (>50 MPa stress).^[^ ^343^ ^]^ By aligning material innovation with clinical pragmatism and regulatory rigor, the field moves closer to achieving durable, biologically integrated restoratives capable of lifelong oral rehabilitation.

## 7 Conclusion and outlooks

The dental materials revolution is transitioning from structural biomimicry to biological integration, where restoratives no longer merely replace lost tissues but actively participate in oral physiological processes. This paradigm is driven by molecular‐scale biological interface programming (such as DNA origami‐guided mineralization), dynamic material ecosystems optimized by artificial intelligence, and closed‐loop biological response systems for perception, adaptation, and regeneration. Our analysis reveals that future breakthroughs may emerge at the intersection of these domains.

Bioinspired strategies, informed by multiscale analyses of dental tissues, have unlocked unprecedented opportunities for engineering materials with tailored mechanical properties, self‐adaptive interfaces, and regenerative capacities. The stress‐redistribution mechanisms of the enamel‐dentin junction, the fluid‐mediated damping of dentinal tubules, and the self‐organized prismatic structure of enamel have inspired innovations such as functionally graded composites, nanofibrous scaffolds, and piezoresponsive ceramics. These materials now approach the biomechanical efficiency of natural teeth, with fracture toughness values exceeding 2.5 MPa·m^1^^/^^2^ and wear rates comparable to enamel's 20–30 µm per year. Moreover, they introduce functionalities such as biofilm resistance, real‐time diagnostics, and controlled drug delivery, blurring the lines between synthetic and biological systems. A particularly innovative direction lies in the development of materials that not only mimic but also augment biological processes—for example, scaffolds engineered to recruit endogenous stem cells or restorations capable of releasing growth factors in response to inflammatory cues, thereby promoting tissue regeneration rather than mere repair.

Advanced manufacturing technologies, especially additive and hybrid methods, have revolutionized the precision and customization of dental restorations. Techniques such as 3D printing of zirconia‐polymer hybrids, 4D‐printed shape‐memory polymers, and two‐photon polymerization enable the fabrication of patient‐specific prosthetics with submicron accuracy. These methods facilitate the integration of multi‐material gradients, enamel‐mimetic microstructures, and stress‐dissipating architectures, enhancing both performance and longevity. Innovations like laser‐induced forward transfer and microwave‐assisted sintering further optimize material properties while reducing energy consumption and waste. However, scalability and cost remain significant barriers, particularly for high‐resolution, multi‐material systems. Addressing these challenges will require industrial partnerships, standardized protocols, and the development of cost‐effective, sustainable manufacturing pathways.

The emergence of smart‐responsive materials signifies a crucial step toward “bio‐smart” dentistry, where materials can actively engage with their biological surroundings. For instance, pH and enzyme‐triggered systems facilitate the targeted release of antimicrobials or remineralizing agents at caries‐prone areas, potentially decreasing the incidence of secondary caries by 60–70%. Self‐healing composites, triggered by occlusal heat or mechanical stress, can restore up to 90% of their original strength, and piezoelectric materials transform chewing forces into antibacterial electrical signals. These advancements illustrate the transition from passive to active materials, although long‐term stability is still a matter of concern. The hydrolytic degradation of responsive polymers and the leaching of nanoparticles call for innovative stabilization techniques, such as atomic layer deposition coatings or covalent adaptable networks. A revolutionary opportunity exists in the incorporation of closed‐loop systems, which, through embedded sensors and actuators, allow for real‐time adaptation to changes in the oral microbiome or biomechanical loads, thus creating restorations that are truly autonomous.

Clinical translation of these advancements faces multifaceted hurdles, including regulatory gaps, in vivo performance variability, and the need for robust evidence platforms. While bioactive materials such as calcium silicate cements achieve high marginal sealing efficiency in vitro, their real‐world performance is often compromised by enzymatic degradation and cyclic loading. Regulatory frameworks must evolve to address dynamic material behaviors and multifunctional integration, ensuring safety and efficacy. Real‐world evidence platforms, which integrate electronic health records with material degradation data, offer a promising pathway but require validation across diverse populations. The success of emerging technologies, such as CRISPR‐engineered bioactive coatings or exosome‐laden hydrogels, will hinge on rigorous longitudinal studies and ethical considerations surrounding biohybrid constructs.

Sustainability has emerged as a critical design criterion, aligning dental materials science with global environmental imperatives. Solvent‐free resins derived from renewable monomers, closed‐loop recycling systems, and the material efficiency of additive manufacturing significantly reduce ecological footprints. However, the environmental impact of nanoparticle synthesis and the end‐of‐life disposal of smart materials remains underexplored. Life‐cycle assessments and circular economy models tailored to dental applications are essential to ensure that innovation does not come at the expense of planetary health.

Looking ahead, the future of dental restoration lies in harmonizing four interconnected pillars: biological fidelity, manufacturing intelligence, dynamic adaptability, and ecological responsibility. The ultimate vision is a future where dental restoratives evolve alongside the patient materials that self‐report degradation via quantum dot tracers, adapt modulus to bruxism forces through machine learning, and even harness oral microbiome metabolites for continuous self‐repair. This will require unprecedented collaboration across disciplines, from synthetic biology to regulatory science, but the payoff is that lifelong, self‐sustaining oral health is within reach.

## Conflict of Interest

The authors declare no conflict of interest.
